# Polydioxanone implants: A systematic review on safety and performance in patients

**DOI:** 10.1177/0885328219888841

**Published:** 2019-11-26

**Authors:** Joana A Martins, Antonina A Lach, Hayley L Morris, Andrew J Carr, Pierre-Alexis Mouthuy

**Affiliations:** 1 Botnar Research Centre, Nuffield Department of Orthopaedics, Rheumatology and Musculoskeletal Sciences, University of Oxford, Oxford, United Kingdom; 2 NIHR Oxford Biomedical Research Centre, John Radcliffe Hospital, Oxford, United Kingdom

**Keywords:** Clinical outcomes, synthetic polymer, medical devices, implants, polydioxanone, safety, performance

## Abstract

**Background:**

Medical devices made of polydioxanone (a synthetic biodegradable polymer) have been available since the early 1980s. However, no review regarding their performance and safety has been published.

**Objective:**

This systematic review intends to review and assess commercially available polydioxanone implants and their safety and performance in patients.

**Methods:**

We searched for approved polydioxanone implants in several Food and Drug Administration databases. Then, we performed a literature search for publications and clinical trials where polydioxanone devices were implanted in patients. This search was performed on MEDLINE, Embase, Scopus and other databases. Safety and performance of polydioxanone implants in patients were assessed and compared with the implantation of non-polydioxanone devices, when possible, based on scoring systems developed by the authors that analyse surgical site infection rates, inflammatory reaction rates, foreign body response, postoperative pain and fever.

**Results:**

Food and Drug Administration databases search revealed that 48 implants have been approved since 1981, with 1294 adverse reactions or product malfunction in the last decade and 16 recalls. A total of 49 clinical trials and 104 scientific publications were found. Polydioxanone sutures and meshes/plates had low rates of surgical site infection, inflammatory reaction, foreign body response and postoperative fever. Polydioxanone clips/staples reported high rates of surgical site infection, postoperative fever and pain, with sub-optimal clinical performance and poor safety rates. The remaining implants identified showed high levels of safety and performance. Safety scores of polydioxanone implants and non-polydioxanone alternatives are similar. Polydioxanone monofilament sutures perform better than non-polydioxanone alternatives but performance did not differ with remaining polydioxanone implant types.

**Conclusions:**

Although polydioxanone clips/staples should be implanted with caution and monitored carefully, in general, safety and performance scores of other polydioxanone implants did not differ from non-polydioxanone alternatives. This review will be a useful reference for researchers and industries developing new polydioxanone medical devices.

## Introduction

Medical devices can be used for diagnosis, treatment, cure or prevention of disease in humans and animals.^1^ A variety of materials can be used in the manufacture of medical devices with consideration to their medical application and material properties, such as strength, stability and degradation profile. Ideal medical implants are affordable and easily available but should also provide good biocompatibility, avoiding any foreign body reaction (FBR).^2^ Medical device implants can take the form of degradable and non-degradable materials, and the former should degrade to nontoxic products after their function has been achieved.^3^ A key advantage of a biodegradable device over a non-degradable device is that it does not require a second surgery for implant removal which reduces the number of hospital visits by the patient, healing time and hospital costs while also avoiding a potential long-term immune response.^4^

Commonly, degradable medical devices are made from polymers which are chains of repeated monomers and can be natural or synthetic. These types of materials are attractive for medical applications due to their ability to tailor mechanical properties and degradation profiles, and the possibility to have designed functional groups for different medical purposes.^3,5^ The small variability between batches and typically reduced chronic FBRs are also an advantage of synthetic polymers when compared to natural polymers,^6,7^ such as silk, cellulose and chitosan. Different polymers are already in use in different clinical applications, including vascular stents, bone cements and wound dressings.^5^

Polyesters are a class of polymers in which their degradation is caused by the hydrolysis of ester linkages, and the degradation products of these types of polymers are resorbed through metabolic pathways.^3^ Some of the most common biodegradable synthetic polyesters used for medical applications include poly(glycolic acid), poly(lactic acid) and poly(caprolactone) which are the main components of common sutures and suture anchors including Safil® sutures, VICRYL® sutures and MONOCRYL® sutures, respectively.^3,5^

**Figure 1.**
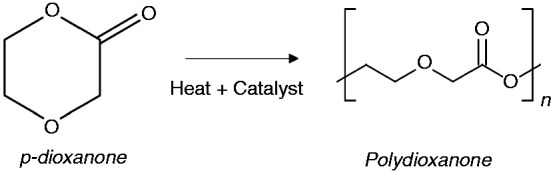
Chemical structure of monomer p-dioxanone and polymer polydioxanone.

Polydioxanone (PDO) is another polyester commonly used for the manufacturing of biodegradable medical devices. Also known as PDS, poly-p-dioxanone, PDX or PDDX, it is a synthetic and absorbable colourless polyester that was first manufactured in the early 1980s.^8^ PDO is commonly synthesised by ring-opening polymerisation of p-dioxanone in the presence of an organometallic catalyst and heat (Figure 1).^9,10^ According to literature provided with existing PDO products, this polymer is considered nonantigenic and nonpyrogenic and is found to induce minimal tissue reaction during absorption after implantation. PDO is degraded by hydrolysis and is completely metabolised in the body.^9^ Due to its relatively prolonged absorption duration (182–238 days) and synthetic nature, PDO is widely used in dyed or undyed medical devices to be implanted where a long-lasting absorbable material is desirable.^11^ PDO is a semi-crystalline polymer (approximately 55% crystalline) with a glass transition temperature ranging between –10°C and 0°C and a melting temperature of 110–115°C.^6,9,10^ These characteristics allow PDO sutures to be manufactured via an extrusion process at the lowest possible temperature, preventing spontaneous depolymerisation.^9^

In the medical field, PDO has been implanted in different shapes and sizes and several PDO medical devices are already used as standard practice in surgeries namely PDS™ II sutures (Ethicon) and OrthoSorb® orthopaedic pins (Johnson & Johnson International). PDO sutures are generally highly flexible due to the presence of an ether oxygen group within the polymer backbone.^9^ However, even though PDO devices are widely used, there has been concern regarding the acidic degradation products released in the body.^4^

To the authors’ knowledge, no publication has provided an overview of the clinical safety and performance of PDO implants to date. In order to aid the development of future PDO medical implants, we review the safety and performance outcomes of CE marked and/or Food and Drug Administration (FDA) approved PDO implantable medical devices and their use in clinical trials or other medical applications.

## Methods

### List of PDO medical implants approved by FDA

We performed a search through the FDA 510(k) and Premarket Approval (PMA) databases in March 2019 using the search terms ‘polydioxanone’, ‘PDO’, ‘PDS’, ‘PDX’, ‘PDDX’ and ‘poly-p-dioxanone’ to find all approved PDO medical devices that are implantable. Inclusion criteria are: implantable medical devices made of PDO with or without antibacterial coating. Exclusion criteria are: non-medical devices, devices not made of PDO, devices made non-exclusively of PDO (excluding antibacterial coating), devices that are not implantable in the human body and devices made of unspecified material. It should be noted that, as discussed in the ‘Marketed medical implants and their classification’ section, clinical data are not mandatory for a premarket notification 510(k) submission. Clearance by FDA to market a medical device using this process is by substantiation of equivalence to other marketed predicate devices; therefore, the FDA 510(k) database may contain medical devices which have not yet been commercialised or used clinically.

After analysis and selection of obtained results, a predicate tree was designed based on a relationship of substantial equivalence provided in each 510(k) submission, when available.

### Manufacturer and User Facility Device Experience Adverse Event Reports

The FDA’s Manufacturer and User Facility Device Experience (MAUDE) Adverse Event Report database was searched for the search terms listed in the ‘List of PDO medical implants approved by FDA’ section by year, from 2008 to 2018. The same inclusion and exclusion criteria apply. Results were also excluded if they were neither adverse event reports nor product problem reports. This database was searched in May 2018 and the number of results was counted by event type and year.

### Medical Device Recalls

The FDA Medical Device Recalls database was searched in May 2018 using the same search terms listed in the ‘List of PDO medical implants approved by FDA’ section and by analysing the information provided with each 510(k) submission. The same inclusion and exclusion criteria apply.

### Clinical studies search strategy and criteria

A review was performed to systematically analyse the clinical safety of PDO medical implants. We performed a search through the Clinical Trials.gov and the International Clinical Trial Platform (ICTRP) websites using the search terms listed in the ‘List of PDO medical implants approved by FDA’ section and adding the brand name of PDO devices found in the FDA search as extra search terms. Four research databases (MEDLINE, Embase, Scopus and Web of Science) were also used. In these research databases, the search terms listed in the ‘List of PDO medical implants approved by FDA’ section were used together with ‘clinical trial’, ‘patient’, ‘human’ and ‘clinical’. In addition, bibliographical references of identified articles were reviewed. Inclusion criteria applied were: clinical trials or studies performed in patients or case reports where PDO medical implants represented at least one of independent variables. Exclusion criteria applied to studies were: ongoing clinical trials with no public results, non-English language publications, studies not performed with human patients, reviews and abstract only publications. These searches were performed in March 2019. Relevant clinical trials and scientific publications that evaluated and compared PDO devices with non-PDO devices were analysed and scored separately based on surgical outcomes (online Appendix 1). A second scoring system was created (online Appendix 2) to analyse all publications that meet the inclusion/exclusion criteria. This second scoring system looked at the percentage of unfavourable outcomes and percentage of successful implants to evaluate safety and performance, respectively. Outcomes were only scored when they were explicitly reported and related to the device. Five major factors were analysed in both scoring systems with regard to material safety: surgical site infection (SSI) rate, presence of inflammatory reaction, presence of foreign body response, presence of postoperative fever and presence of postoperative pain. Performance levels were evaluated separately based on the success of implants.

## Results

### PDO devices with regulatory approval

A number of 81 submissions were reported from the 510(k) database, with only 47 being admissible and relevant to this study. The remaining 34 submissions referred to medical devices that either had other polymers in their composition, were not implanted in the body or insufficient information was provided to meet requirements. A list of admissible devices can be found in online Appendix 3.

The only PDO device found in the PMA database was PDS suture from Ethicon (PMA number N18331) with supplements from S001 to S027. These PMA supplements are based on changes to the original device and can include changes in the device itself, its packaging or labelling or its manufacturing process. The name of this suture was changed to PDS II with supplement S022 dated from 1990.

**Figure 2.**
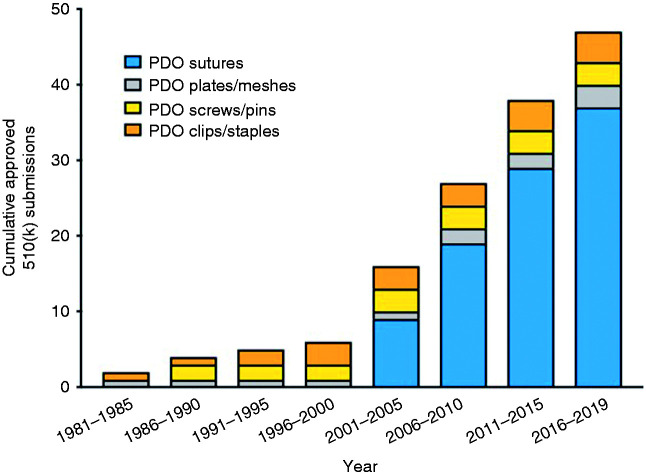
Cumulative number of 510(k) submissions of different types of PDO medical devices.

Based on the search results described, PDO medical implants can be distributed into four main categories: sutures, plates/meshes, clips/staples and screws/pins (online Appendix 3). Based on these data, an evolution of the approved PDO medical implants can be seen in Figure 2. The higher number of approvals over time shows there is an ongoing and an increased interest in PDO implants and their use in patients since PDO was first introduced in 1981.^8^

Based on the available information on admissible 510(k) submissions, a predicate tree was designed (online Appendix 4). Each device is linked to its predicates and to the devices they are predicates to, on the basis of substantial equivalence. Due to limited information provided by the 510(k) database, predicates are unidentified for nine submissions. The majority of FDA approved PDO devices are sutures and are linked directly or indirectly to PDS™ II which was approved via PMA route. Based on the information available, at least six submissions involve sutures that differ from their predicate based on minor modifications (for instance, additional suture size). Moreover, 10 implants are considered substantially equivalent to non-PDO devices in a direct way, with six of them also being considered substantially equivalent to other PDO devices.

Shape, surface area and weight of an implant can affect how the body reacts to it, even if the core material is the same.^12^ For that reason, these factors should be assessed when analysing different types of medical devices. In Table 1, it is possible to see the differences in dimensions and weight of common PDO devices. We calculated the estimated weight of each based on an estimated PDO density of 1.318 g/cm^3^.^13^ It should be noted that the estimated weight is per device and does not necessarily correspond to the weight of PDO that is implanted in the body during surgery, dependent upon device application.

**Table 1.** Dimensions of different types of PDO devices, their estimated weight and representative figure.

PDO device	Type of device	Product code	Dimensions	Estimated weight	Representative figure
PDS™ II	Monofilament suture	Z997G	USP 2-070 cm length^11^	0.2 g	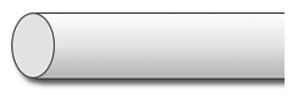
PDS™ Flexible Plate	Unperforated plate	ZX5	0.25 × 40 ×50 mm^14^	0.7 g	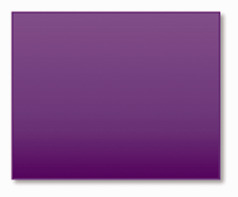
OrthoSorb®	Straight pin	84-2052	Ø 2 mmlength 40 mm^15,16^	0.2 g	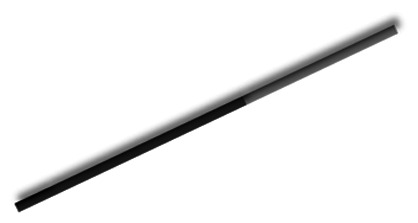
Lapra-Ty®	Clip	XC200	0.3 cm beam length0.3 cm width^17^	0.1 g	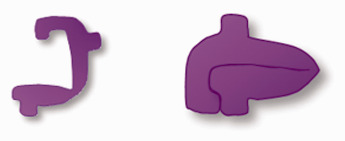 Open Closed

Note: Basic information regarding the characteristics of the different implant types can be found in online Appendix 5.

### MAUDE Adverse Event Reports

**Figure 3.**
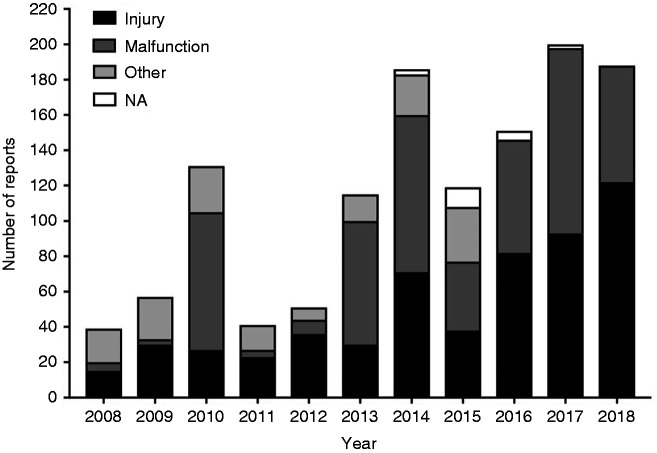
Manufacturer and User Facility Device Experience results by year reporting adverse events and product problem reports involving PDO implants. NA: not available.

The MAUDE database revealed 6859 results. After applying the inclusion and exclusion criteria, 1377 results were admissible. After removing duplicates, the search totalled 1278 results identifying adverse event reports and product problem reports. Figure 3 represents the number of adverse event reports and product problem reports related to PDO devices from January 2008 to April 2018 (included). As it can be seen, the main reason for adverse events and product problems is injury and malfunction. No deaths were reported as being caused by PDO implants.

### Medical Device Recalls

The FDA Medical Device Recalls database provided 24 results. After applying the inclusion/exclusion criteria and removing duplicates, 16 recalls involving PDO devices were deemed admissible. These are presented in Table 2.

**Table 2.** PDO Medical Device Recalls.

Trade name	PMA/510(k) number	FDA recall posting date	Recall number	Recall class	Reason for recall
Absolok	K812323	2005	Z-0654-05	2	Possible sterility issue
		2005	Z-0655-05	2	Possible sterility issue
		2005	Z-0656-05	2	Possible sterility issue
		2005	Z-0657-05	2	Possible sterility issue
Monodek	K030212	2007	Z-1016-2007	2	Possible sterility issue
		2014	Z-1509-2014	2	The product did not meet minimum knot tensile strength requirements
		2014	Z-1534-2014	2	The product did not meet minimum and/or average minimum resorption strength requirements
Mono-Dox	K013274	2007	Z-0809-2007	2	Possible sterility issue
		2008	Z-2582-2010	2	Possible compromise of seal integrity of the inner product pouch
		2009	Z-0259-2012	2	Possible compromise of seal integrity of the outer product pouch
PDS™ II	N18331	2009	Z-1313-2009	2	Possible sterility issue
PDS™ Plus	K061037	2009	Z-1314-2009	2	Possible sterility issue
		2018	Z-1338-2018	2	Incorrect expiry date on the label
		2018	Z-1339-2018	2	Incorrect expiry date on the label
		2018	Z-1340-2018	2	Incorrect expiry date on the label
PDS™ Barbed Suture	K113004	2013	Z-0458-2014	2	The product has a small number of tab failures and fascial dehiscences in lower abdominal incisions

PDS™ Barbed suture (K113004) was recalled in 2013 due to a small number of tab failures and fascial dehiscences in lower abdominal incisions, while the others were recalled due to packaging and sterility issues or by not meeting the product specifications. It is evident from the data that none of these devices were recalled due to problems with device material.

### Clinical trials and clinical studies

After removal of duplicates and application of the inclusion/exclusion criteria, 153 publications involving clinical studies were deemed acceptable and analysed. Of the admissible publications, 49 were clinical trials with published results and 104 were scientific publications. Scientific publications include retrospective studies and case reports. Publications show that there is a high report of PDO implants in Trauma and Orthopaedic Surgery, Otolaryngology and General Surgery.

**Figure 4.**
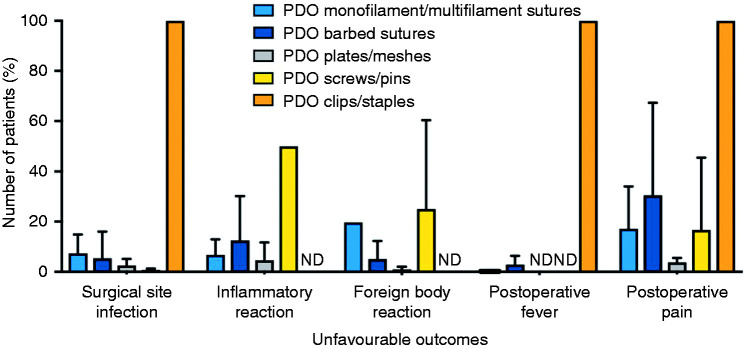
Percentage of unfavourable outcomes of PDO implants according to type of implant. ND: no data.

**Figure 5.**
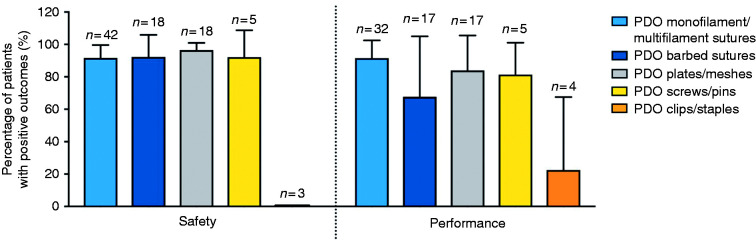
Safety and performance efficacy of PDO implants. n: number of publications.

Scientific publications and clinical trials were scored according to online Appendices 1 and 2. While using the scoring system, 11 publications and clinical trials did not comment on any of the assessed five unfavourable outcomes and performance and as such were removed from analysis. When comparing the different types of PDO implants (Figure 4), the highest percentage of unfavourable outcomes was reported for clips and staples, resulting in lower safety scores. Figure 5 shows that PDO devices have over 90% safety and over 65% performance efficacy, with the exception of PDO clips and staples.

**Figure 6.**
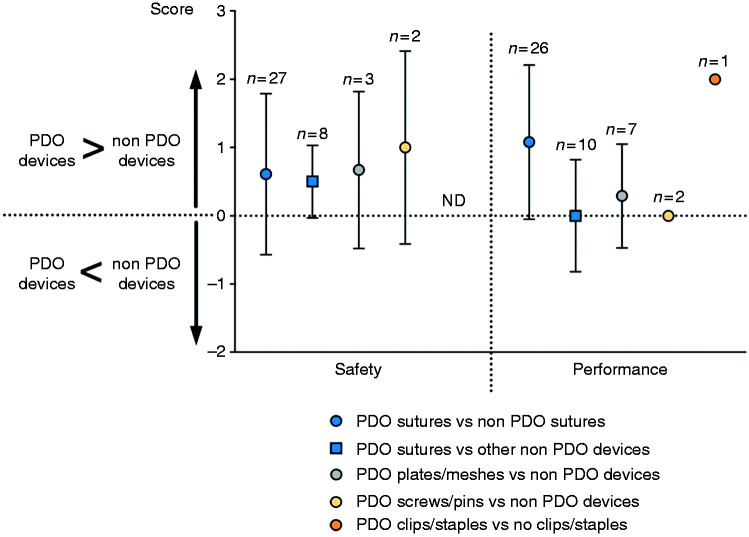
Comparison of PDO implants with non-PDO implants regarding safety and performance efficacy. n: number of publications; ND: no data.

Figure 6 compares safety and performance of PDO implants with non-PDO implants according to online Appendix 1. With regard to safety, PDO implants generally have higher safety scores than non-PDO repairs. With regard to performance, in general it did not differ when compared to non-PDO devices.

## Discussion

### Marketed medical implants and their classification

The U.S. FDA regulates all medical devices marketed in the United States since 1976. The FDA process aims to ensure all devices are safe and effective, from development to distribution and use.^18^ The 1976 Medical Device Amendments separated medical devices into regulatory classes based on their degree of risk: Class I, Class II and Class III, where the latter include the most complex and highest risk devices. This classification process is similar to the one that the European Commission has in place in Europe, as defined by the 2017/745 Medical Device Regulation. Most Class II devices are required to submit a premarket notification (510(k)), while most of Class III devices require PMA before they are marketed. While Class III devices have the most stringent level of regulation, they may be marketed through the 510(k) route if they are substantially equivalent to any other devices marketed before 28 May 1976, unless the FDA requires PMA data.^18^ With the 510(k) premarket notification route, manufacturers have the opportunity to obtain a faster market approval as long as they can prove their device is substantially equivalent to a device that is already legally marketed. This marketed device is called the predicate if equivalence claims are substantiated. This substantial equivalence is based on intended use, technological characteristics, and safety and efficacy. In order to demonstrate equivalence, animal data or human clinical trial results may be included as part of the 510(k) submission. However, it should be noted that approval to market via the 510(k) route does not necessarily mean there is clinical data available or that the device was used previously in other markets, such as in Europe. On the other hand, PMA requires more detailed information, namely clinical protocols, adverse reactions and complications, patient complaints, technical data and non-clinical data (e.g. toxicology, tensile strength and shelf life).^18^ Additions to the 1976 Medical Device Amendments were later introduced in the 1990s, including the Medical Device Reporting, which made possible for the FDA to receive information about significant medical device adverse events. In addition, the FDA has to make publicly available the FDA 510(k) premarket reviews following the Safe Medical Devices Act of 1990.^18^

As presented earlier, 47 PDO implants were approved via 510(k) route and only one implant was approved via the PMA process (online Appendix 4). This PMA approved device (PDS™ suture, approved for commercialisation in 1982) was considered, directly or indirectly, as a predicate to the majority of PDO sutures subsequently approved via 510(k). Thus, over 97% of PDO sutures’ submissions (all approved on the basis of substantial equivalence) were not necessarily required to detail comprehensive information about the product, such as non-clinical data and/or animal/clinical data, unless specified by the FDA. This kind of approach may cause a problem if the predicate causes adverse reactions in clinical use post market approval that can lead to product recall. However, this does not necessarily mean medical devices that reference the recalled product as predicate would also be under investigation. Additionally, 10 PDO implants were found to have non-PDO devices as predicates directly, namely on the basis of similar function or shape. Suppliers of these implants might not be considering the combined impact of material and shape for the desired function in these submissions, and their influence on the degradation process and host tissue reaction after implantation. Since animal and/or clinical data may not be required for 510(k) submission, host tissue reaction and possible adverse reactions would not be assessed until the device is implanted in patients after market approval. Finally, since nine PDO medical implants have no publicly available data regarding their predicates, it is not possible for authors to assess predicate suitability.

As explained in the ‘PDO degradation process’ and ‘FBR and inflammatory reaction’ sections, implant mass can greatly impact biocompatibility. Table 1 shows that a PDS plate weighs more than comparative PDO implants and that Lapra-Ty® had the lowest weight and dimensions. Shape, dimensions, surface characteristics, weight of the device and mechanical integrity can influence how the host tissue reacts with the different implants, even though they are manufactured from the same material.^12^ For that reason, these parameters need to be taken into account when designing an implant.

The effect of approved PDO implants in patient safety and efficacy is further discussed in the ‘Safety scoring system – Measured outcomes’ section.

### MAUDE Adverse Event Reports

MAUDE database is limited to adverse event reports within the past 10 years. This database consists of all voluntary, user facility, distributor and manufacturer reports since early 1990s. It is the manufacturers’ and importers’ responsibility to submit reports when they become aware of information that reasonably suggests that one of their medical devices contributed to a death or serious injury or has malfunctioned.

When examining Figure 3, there is a high number of malfunction and injury reports (1278 reports) for the different types of PDO implants but this is a small percentage when considering the number of patients that have PDO devices implanted each year (in comparison, in the same time period, over 4000 reports were found for VICRYL® sutures – made of polyglactin 910 – alone). In addition, it is not possible to say that these reported incidents were caused by the medical device material or shape rather than as result of the surgical procedure or technique used. This is due to the fact that, in the majority of cases, it was not possible to recover the medical device for investigation, or further information was not available or provided by FDA. None of these adverse events and product problem reports claimed that PDO devices were related to any patient death.

### Medical Device Recalls

Medical Device Recalls occur when a device is defective and/or presents a risk to health. Sometimes this means the device just needs to be checked, adjusted or fixed and does not mean a product is discontinued or withdrawn from the market. Recalls can be categorised into three different categories: Class I (there is a reasonable chance that a product will cause serious health problems or death), Class II (product may cause a temporary or reversible health problem or there is a slight chance it will cause serious health problems or death) and Class III (product is not likely to cause any health problem or injury). The FDA Medical Device Recalls database contains recall reports dating back to November 2002.

As it is possible to see from Table 2, none of these devices were recalled due to device material, as the chemistry of the device could not have influenced the seal or packaging integrity nor the meeting of final specifications, as this is part of the quality control process of each product during manufacturing. Regarding PDS™ Barbed Suture’s recall, fascial dehiscence may have been caused by the barbs on the suture, rupturing the fascia along the surgical incision. There is no information if this product is still on the market and the product cannot be found in an online search.

### PDO degradation process

It is important to understand the biodegradation process of PDO when looking at biocompatibility and the use of PDO implants. The degradation process of a biodegradable implant should ideally match the healing rate of the tissue where it is implanted, thus maintaining the device’s mechanical properties while they are required.^7^ The degradation process can occur after implantation but also start during manufacturing while PDO is being melted and/or extruded into the desired shape. This initial degradation can lead to a decrease of molecular weight and as such moisture and high temperature should be minimised during manufacturing to prevent changes in the polymer’s properties and further impact product shelf life.

Hygroscopic polymers like PDO can be hydrolysed, resulting in degradation by-products such as glycoxylate that can be excreted in urine, or be metabolised via citric acid cycle to form carbon dioxide and water.^9,19^ Several authors also reported oxalic acid, propylene glycol, 2-hydroxyacetic acid and glyoxylic acid as degradation products.^20^ The release of glyoxylic acid can contribute to a chronic inflammatory response due to a local decrease in pH.^21,22^ Additionally, this acidic environment can be caused by a sudden and rapid release of degradation products or by a large amount of material present or large surface area.^4^ When compared on a weight basis to other polymers such as PLA and PGA, PDO degradation products are less acidic, which could cause less inflammatory reaction.^21^

Biomaterial degradation is affected by the surrounding area and tissue, namely by the mechanical forces involved, disease or healthy state and vascularisation.^23^ Microstructural cracks created by a high-stress environment can lead to an enhanced exposed surface area and to a loss of mechanical strength in addition to any chemical reactions that may occur simultaneously.^19^ During the early stages of degradation, the water penetrates the bulk of the implant, breaking the chemical bonds in the amorphous phase.^6,7^ This step cuts down long polymer chains, reducing the average molecular weight and increasing polydispersity. At this point, the implant’s gross material properties are still maintained as they are defined by the crystalline regions, which are unaffected.^6^ Decrease of average molecular weight usually occurs before any material loss is observed.^24^ At the last stage of degradation, the water hydrolyses the small water-soluble fragments which leads to loss of polymer mass, molecular weight and physical properties.^6,25–27^ The resulting degradation products are then quickly removed from the surface in the surrounding fluid. However, in the interior of the device, the inability to diffuse degradation products creates an acidic environment that will then lead to an accelerated hydrolysis of the ester bonds. This process explains why a low porosity implant may degrade faster than a high porosity implant, having a lower functional lifespan.^4,6,19,25,26,28^

Biomaterial degradation is also dependent on basal metabolic rates of the test subjects, with degradation rates in human being slower than in laboratory animals.^15,29^ In vitro studies using PDO sutures showed that after 10 weeks in a buffer solution, sutures still retained over 96% of their weight.^26^ While testing PDS™ II sutures in vitro, Ooi and Cameron^27^ also observed that there is an initial hydration of the material during the first two days, followed by a dormant stage up to 20 days when scission of the tie chain initiates. An active stage then follows, in up to 60 days, when there is a decrease in tensile breaking strength and strain, and an increase in water uptake. Lin et al. reported a loss of around 67% tensile strength in 14–28 days of hydrolysis using PDS™ sutures in PBS buffer, with low loss of tensile strength in 42–60 days.^7^ However, with regard to mass absorption, only 1.5% of mass was absorbed at 60 days, while 90% of tensile strength was already gone. In some cases, this material left after the suture has achieved its purpose may cause FBR and granuloma formation^7^ but this outcome will disappear once the material is fully degraded. Complete absorption of PDS™ sutures has been reported from 117 to 240 days, with 67% loss of tensile strength at 14–28 days postimplantation.^7^ PDO clips can take more than 180 days to fully degrade.^20^ PDO pins have also been reported as 80% being visible at three and six months post implantation in an animal model, with total resorption by 24 months.^15^ In addition, in vitro toxicity of degradation products may not match in vivo biocompatibility. In an in vivo study, 93% of PDS™ sutures’ degradation products were removed in urine.^8^ Other studies have also indicated that PDO has good biocompatibility and a positive biological safety in rabbits.^30^

### Safety scoring system – Measured outcomes

SSIs, FBR, inflammatory reaction, postoperative pain and fever have been analysed in clinical trial results and scientific papers that reported the use of PDO implants, based on the shape and type of the device. There is no uniform assessment across the different papers since authors evaluated different outcomes. For that reason, postoperative fever and pain were considered as separate outcomes from SSI and FBR because authors may not explicitly define SSI and FBR as the cause for postoperative fever and pain. Based on these outcomes, we developed two scoring systems: one for the evaluation of PDO implants based on the percentage of patients with adverse reactions reported (online Appendix 2) and another scoring system comparing PDO with non-PDO implants (online Appendix 1). In this section, we explain how the five mentioned outcomes can be influenced by implant material.

#### Surgical site infections

According to Gabriel et al., in the U.S. around two million people are infected each year due to hospital stays, causing 90,000 yearly deaths.^31^ SSI rates can be affected by different factors including general condition of the patient (such as age, fitness and comorbidities), the presence of pathogenic bacteria, lack of pre-operative skin disinfection, contaminated surgical instruments and nosocomial infections acquired secondary to prolonged hospital stays.^32^

Implantable medical devices can also be a cause of SSI (known as implant-related infection) due to bacterial colonisation and a high and continuous implantation time. This bacterial colonisation could lead to the formation of a multilayer biofilm which could result in failure of the implanted device and its consequential removal. This biofilm could also lead to high resistance to host defence mechanisms and antibiotic treatment.^32^ The material surface characteristics are very important in bacterial adhesion, namely the surface chemical composition, surface roughness, surface configuration and exposed area, when interacting with the bacterial properties (bacterial hydrophobicity and bacterial surface charge).^32^ Medical devices, particularly sutures, have evolved to try to prevent high infection rates through the addition of antimicrobial coatings; however, conflicting information has been found regarding their efficiency.^33–35^ Medical device roughness and configuration play a major role in bacterial adhesion, as several studies showed that bacterial adhesion is promoted by surface irregularities.^36,37^ This suggests that multifilament sutures may be more prone to causing implant-related infection due to their higher surface area when compared to monofilament sutures.^38,39^ However, chemical composition can also affect bacterial adhesion: in vitro and animal experiments showed that implants made of polymethyl methacrylate (PMMA) have been found to be more prone to cause infection, as opposed to titanium and cobalt–chromium implants,^37,40,41^ which can be related to material properties such as surface charge and hydrophobicity, as discussed previously.

Figure 4 shows that PDO sutures, meshes/plates and screws/pins had low SSI rates (below 10%) possibly due to low porosity and smooth exposed area. With regard to PDO clips/staples, SSI was reported in two case reports. Brusky and Tran^42^ reported that a patient showed urinary tract infection five weeks after an uneventful right laparoscopic transperitoneal Anderson-Hynes dismembered pyeloplasty when Lapra-Ty® clips were used to anchor polyglactin sutures. Further study confirmed that Lapra-Ty® clips had migrated into the ureter, possibly being the cause of the urinary infection described. Finley et al.^43^ also reported Lapra-Ty® clip migration in one patient, which could also have caused the reported infection.

#### FBR and inflammatory reaction

Inflammatory response and FBR are expected tissue reactions following implantation of biomaterials.^44^ An inflammatory reaction is defined as the reaction of vascularised living tissue to local injury and can still be initiated even if the implanted material is nontoxic, nonimmunogenic and chemically inert.^45,46^ An inflammatory response can be divided into two stages: acute and chronic inflammation. Each stage is characterised by specific cell types, duration and overall biological importance.^47^ This process has been extensively studied and reviewed.^45,48,49^ Acute inflammation can be caused by tissue damage elicited when a medical device is implanted, and is part of the normal wound-healing process. During this stage, blood proteins (such as albumin, antibodies and fibrinogen) are adsorbed onto the medical device surface.^46,47^ Chemical and physical properties (such as surface chemistry and hydrophobicity) of the device will greatly influence the composition of the adsorbed protein layer. It is the presentation of the various plasma proteins on the material’s surface that creates a high affinity matrix for the subsequent attachment and activation of a range of inflammatory cells, and not the material itself.^47,48^ In addition, material charge may directly affect macrophage and polymorphonuclear neutrophil adhesion.^48,50^

With long-term or permanent non-degradable implants, the inflammatory response generally evolves into a chronic reaction,^51^ thus changing the cellular population surrounding the implant. At this stage, monocyte-derived macrophages are the dominant cell type and will try to break down, encapsulate and remove the implanted material while remodelling the surrounding extracellular matrix.^47^ With biodegradable implants, chronic inflammation is a possibility in some cases and it can accelerate degradation due to the release of hydrolytic enzymes and reactive oxygen and nitrogen species, which reduces the polymer molecular weight.^47^ This way, chronic inflammation could negatively affect the efficacy of implanted devices in permanent or long-term applications. It is worth noting that this reaction will be limited in time and affected area when using biodegradable materials. While the implant is being resorbed, inflammation and FBR will gradually decrease until it disappears.^44,51,52^ Several approaches have been developed to target the issues of inflammation and medical devices including the use of bioinert surfaces and bioactive strategies.^47,53^

The implantation of medical devices can also induce a primary reaction of the nonspecific immune system to foreign materials known as FBR. Usually, the FBR is the end-stage response of the inflammatory and wound healing responses but they can happen simultaneously.^44^ The FBR consists of material-dependent and material-independent processes, and can be induced when local tissues are not able to clear polymeric hydrolytic debris.^23,44^ Usually, synthetic materials do not elicit a specific biological reaction.^45^ The FBR to biomaterials involves foreign body giant cells and components of granulation tissue. Surface properties, shape of the implant and the relationship between surface area and volume of the implant affect the composition of the FBR. For instance, high surface area-to-volume ratio implants and porous materials such as plates and meshes will have an increased number of macrophages and foreign body giant cells at the implant site. On the other hand, smooth surface implants like monofilament sutures will result in more fibrotic tissue at the implant site. During the early stages of inflammation and wound healing, macrophages are activated upon adherence to the implant surface due to the device’s non-native chemical and physical properties.^45^ Adherent macrophages and foreign body giant cells can then lead to the degradation of biomaterials and possible device failure.^44^ FBR can also lead to suture extrusion and rejection and stitch abscesses if a bulky material is present. This transepidermal elimination is aided by superficial suture placement and increased mass of suture materials.^27,54^ FBR may persist at the tissue–device interface for the lifetime of the implant, but after resorption only connective tissue remains.^45^

Figure 4 shows that inflammatory reaction and FBR rates were below 20% for barbed and monofilament/multifilament sutures and below 5% for PDO plates/meshes. Even though PDO plates are the bulkiest device, they reportedly caused FBR in a smaller percentage of patients than other devices, which was not anticipated since porous and bulkier devices are expected to have a higher number of associated macrophages and foreign body giant cells. This difference could be related to the high vascularity and low mobility of the host tissue where these devices were implanted, for instance, during nasal reconstruction.^14,55–57^ Regarding PDO screws/pins, Kalla and Janzen^23^ described an FBR and inflammation in one patient caused by Orthosorb®. This type of PDO pin had been implanted in both feet during separate surgeries for bunionectomy. FBR and inflammation was only observed on the right foot after the second surgery. Kalla and Janzen^23^ suggested that the observed FBR could have been caused by low vascularity and temperature at the implantation site, and also due to a possible immunological sensitisation to Orthosorb® after the first surgery. However, it is worth noting that this was the only reported case of FBR with Orthosorb(R) in the available publications. No paper reported the rate of inflammatory reaction or FBR after the implantation of PDO clips/staples. It is also worth noting that only 0.2% patients reportedly had FBR in the 142 clinical studies analysed.

#### Postoperative fever

The reported incidence of postoperative fever varies, but can be expected in 13–14% of patients.^58,59^ Figure 4 shows that postoperative fever was below 4% in monofilament/multifilament and barbed sutures, which is better than reported rates. PDO clips had a high postoperative fever caused by clip migration as discussed previously. Postoperative fever assessments following the implantation of PDO plates/meshes and screws/pins were not reported in any of the publications reviewed. It is worth noting that, when analysing clinical trial results and scientific publications, there is no distinction in data analysis between a fever that develops within the first 48 h after surgery (which is usually benign and self-limiting and may be procedure-related) and fever that develops later on (which is more likely to have an infectious cause).^58–60^ The interruption of the normal host defence mechanisms, such as medical device implantation, is a risk factor for development of other comorbidities that can lead to fever.^60^

#### Postoperative pain

Postoperative pain was reported in less than 4% patients implanted with PDO plates/meshes, which is expected since this type of implant reported low levels of SSI, inflammatory reaction and FBR. However, pain levels were higher for monofilament/multifilament sutures (17%), barbed sutures (31%) and screws/pins (17%). Barbed sutures could be causing more local pain than monofilament sutures due to their barbs that attach to the surrounding tissue to keep the suture in place.

### Surgical outcomes from clinical trials and scientific publications

When analysing the percentage of unfavourable outcomes (Figure 4), clinical studies revealed a low percentage of SSI and postoperative fever for PDO sutures, plates/meshes and screws/pins. FBR percentage was higher for PDO screws/pins, followed by monofilament/multifilament sutures and then barbed sutures. Figure 4 also shows that PDO clips/staples have a high rate of SSI, postoperative fever and postoperative pain. These safety results were solely based on three publications of case reports of Lapra-Ty® suture clips, totalling three patients that had history of pyelonephritis (inflammation of the kidney). In these three case reports, clip migration was observed, causing the pain and fever reported. As suggested by other work,^61^ case reports analysed in this review may not be representative of the use of these suture clips. This also implies that it was not the fact that these devices are made of PDO that caused this biological reaction, but a failure in device function. However, this needs to be investigated further. Only one study^62^ was found which reported good performance with Lapra-Ty® clips.

Overall, PDO implants are safe in over 90% of cases and perform as expected in over 80% of cases for sutures, plates/meshes and screws/pins. PDO barbed sutures have a lower performance efficacy rate (67%) and when analysing clinical studies reporting the use of this type of PDO sutures, two case reports^63,64^ described failure of performance due to barb attachment to adjacent tissue and surgical approach and confounding comorbidities, making it clear that in these cases, the performance of the barbed suture was not influenced by the material used.

Figure 6 compares safety and performance of PDO implants with non-PDO devices. Results show that PDO implants show a trend of better safety levels than non-PDO devices. In some cases, PDO devices are compared to non-degradable implants, which can cause long-term FBR, decreasing their safety rates. No publication was found reporting safety outcomes involving PDO clips/staples. Regarding performance, PDO sutures perform better than non-PDO sutures but perform just as well when compared to meshes or surgical glues. It is worth noting that from the 25 papers that assessed suture performance, seven of them compared PDO barbed sutures with non-PDO monofilament sutures. With barbed sutures, the barbs attach to the tissue, locking the suture in place and preventing the incision from opening, which generally increased the performance values of PDO sutures compared to non-PDO smooth monofilament sutures. PDO plates/meshes also show a trend of increased performance levels when compared to non-PDO repair.

PDO screws/pins present similar performance when compared to the non-PDO alternatives but should be noted that only a small number of clinical studies reported performance for these types of devices. Only one publication was found comparing PDO clips/staples (Lapra-Ty® clips) to non-PDO repair, resulting in a score of 2 for performance. From this low number of publications, it is not possible to infer the general performance of these types of devices, since that will also depend on the application of the device and the way it is used by surgeons.

### Future of medical implants

PDO implants have been traditionally manufactured through extrusion, but different techniques can be used while considering the different aspects of designing implants mentioned previously. The manufacturing process should also be taken into account to minimise changes in mechanical properties (unless these are desired for intended use), in particular if the main component is a hydrolytic material. PDO implants should be manufactured with the aim to reduce humidity whenever possible in order to not accelerate degradation.^6^ Excessive high temperatures during manufacturing can also change the mechanical properties of PDO.^65^ For these reasons, manufacturers should not sterilise PDO medical devices by autoclaving or dry heat, but should consider ethylene oxide since this polymer is also sensitive to gamma radiation. Additives can be used during manufacturing to scale up the process or to reduce degradation but care must be taken regarding the potential impact to biological safety.^6^ This review assumes that no additives were left in PDO implants.

### Limitations to this study

It is worth noting that FDA claims that Medical Device Reports data alone cannot be used to conclude about the existence, severity or frequency of problems associated with a device and that establishing relationships between devices and adverse events is especially difficult if circumstances surrounding the event have not been verified or if the device has not been directly evaluated. Clinical studies were analysed in this paper to provide more information regarding safety of this type of implants. We assessed all relevant publications, but we are aware that the number of patients in each clinical publication may not be representative of the general population with implanted PDO devices. Furthermore, when comparing implanted devices made with different materials, the different degradation rate (if applicable) and implantation site may affect the observed results.

In addition, despite a medical device being approved by the FDA, it does not mean it is currently being sold in the market. Moreover, medical device technical information, such as degradation profile, molecular weight, animal tests performed, is not available due to confidentiality. This lack of transparency makes it harder to assess the medical device as a whole during its application in the human body.

## Conclusion

PDO medical implants are widely used in a number of different surgical specialties with varying shapes, sizes and configurations. The results of this review show that no product has been recalled due to the material used and that no PDO implant caused a patient death. In addition, the low percentage of adverse reactions from the use of PDO implants in patients shows that PDO sutures, plates/meshes and screws/pins are safe to use and perform as expected. Care must be taken when using PDO clips/staples so that they are applied correctly since failure in function can cause PDO clips to migrate and cause adverse reactions. When compared to non-PDO repair, PDO implants are generally safe and perform at least as well as alternatives.

This review suggests that, as a biodegradable polymer and while considering the biological properties of the host tissue and the shape and dimensions of the device, PDO is a safe material to use in the development of further and innovative biodegradable medical implants.

## Supplemental Material

JBA888841 Supplemental Material1 - Supplemental material for Polydioxanone implants: A systematic review on safety and performance in patients Supplemental material, JBA888841 Supplemental Material1 for Polydioxanone implants: A systematic review on safety and performance in patients by Joana A Martins, Antonina A Lach, Hayley L Morris, Andrew J Carr and Pierre-Alexis Mouthuy in Journal of Biomaterials Applications

JBA888841 Supplemental Material2 - Supplemental material for Polydioxanone implants: A systematic review on safety and performance in patients Supplemental material, JBA888841 Supplemental Material2 for Polydioxanone implants: A systematic review on safety and performance in patients by Joana A Martins, Antonina A Lach, Hayley L Morris, Andrew J Carr and Pierre-Alexis Mouthuy in Journal of Biomaterials Applications

JBA888841 Supplemental Material3 - Supplemental material for Polydioxanone implants: A systematic review on safety and performance in patients Supplemental material, JBA888841 Supplemental Material3 for Polydioxanone implants: A systematic review on safety and performance in patients by Joana A Martins, Antonina A Lach, Hayley L Morris, Andrew J Carr and Pierre-Alexis Mouthuy in Journal of Biomaterials Applications

JBA888841 Supplemental Material4 - Supplemental material for Polydioxanone implants: A systematic review on safety and performance in patients Supplemental material, JBA888841 Supplemental Material4 for Polydioxanone implants: A systematic review on safety and performance in patients by Joana A Martins, Antonina A Lach, Hayley L Morris, Andrew J Carr and Pierre-Alexis Mouthuy in Journal of Biomaterials Applications

JBA888841 Supplemental Material5 - Supplemental material for Polydioxanone implants: A systematic review on safety and performance in patients Supplemental material, JBA888841 Supplemental Material5 for Polydioxanone implants: A systematic review on safety and performance in patients by Joana A Martins, Antonina A Lach, Hayley L Morris, Andrew J Carr and Pierre-Alexis Mouthuy in Journal of Biomaterials Applications

JBA888841 Supplemental Material6 - Supplemental material for Polydioxanone implants: A systematic review on safety and performance in patients Supplemental material, JBA888841 Supplemental Material6 for Polydioxanone implants: A systematic review on safety and performance in patients by Joana A Martins, Antonina A Lach, Hayley L Morris, Andrew J Carr and Pierre-Alexis Mouthuy in Journal of Biomaterials Applications

JBA888841 Supplemental Material7 - Supplemental material for Polydioxanone implants: A systematic review on safety and performance in patients Supplemental material, JBA888841 Supplemental Material7 for Polydioxanone implants: A systematic review on safety and performance in patients by Joana A Martins, Antonina A Lach, Hayley L Morris, Andrew J Carr and Pierre-Alexis Mouthuy in Journal of Biomaterials Applications

JBA888841 Supplemental Material8 - Supplemental material for Polydioxanone implants: A systematic review on safety and performance in patients Supplemental material, JBA888841 Supplemental Material8 for Polydioxanone implants: A systematic review on safety and performance in patients by Joana A Martins, Antonina A Lach, Hayley L Morris, Andrew J Carr and Pierre-Alexis Mouthuy in Journal of Biomaterials Applications
