# Supplementary material for: Polydioxanone implants: A systematic review on safety and performance in patients
Source: J Biomater Appl. 2019 Nov 26;34(7):902–16. doi: 10.1177/0885328219888841 (PMC7044756; doi:10.1177/0885328219888841)
Supplement: JBA888841 Supplemental Material1 - Supplemental material for Polydioxanone implants: A systematic review on safety and performance in patients [file JBA888841_Supplemental_Material1.pdf]

Appendix 1 - Scoring system based on a comparison between PDO implants and non PDO implants or repair.

Outcomes				Score
Safety	Surgical Site Infection (SSI)	Reported no SSI in PDO group		2
		Reported SSI in PDO group:		
			SSI rates lower in PDO group	1
			Similar rates of SSI	0
			SSI rates higher in PDO group	-2
		Not commented / not assessed		-
	Inflammatory reaction	Reported no inflammatory reaction in PDO group		2
		Reported inflammatory reaction in PDO group:		
			Lower inflammatory reaction in PDO group	1
			Similar inflammatory reaction	0
			Higher inflammatory reaction in PDO group	-2
		Not commented / not assessed		-
	Foreign body reaction	Reported no foreign body reaction in PDO group		2
		Reported foreign body reaction in PDO group:		
			Foreign body reaction is lower in PDO group compared to non PDO group	1
			Foreign body reaction is similar in all groups	0
			Foreign body reaction is higher in PDO group compared to non PDO group	-2
		Not commented / not assessed		-
	Postoperative fever	Reported no postoperative fever in PDO group		2
		Reported postoperative fever in PDO group:		
			Postoperative fever rate lower in PDO group	1
			Similar rates of postoperative fever	0
			Postoperative fever rate higher in PDO group	-2
		Not commented / not assessed		-
	Postoperative Pain	Reported no pain in PDO group		2
		Reported postoperative pain in PDO group:		
			Postoperative pain scores lower in PDO group	1
			Similar postoperative pain scores	0
			Postoperative pain scores higher in PDO group	-2
		Not commented / not assessed		-
Performance		Performance of medical device assessed in PDO group/device(s):		
			PDO device performs as expected and with better performance than comparative device	2
			PDO device performs as expected and similarly as comparative device	0
			PDO device performs as expected but performance of comparative device is better	-1
			PDO device fails to meet expected performance	-2
		Not commented / not assessed		-
