# Supplementary material for: Polydioxanone implants: A systematic review on safety and performance in patients
Source: J Biomater Appl. 2019 Nov 26;34(7):902–16. doi: 10.1177/0885328219888841 (PMC7044756; doi:10.1177/0885328219888841)
Supplement: JBA888841 Supplemental Material2 - Supplemental material for Polydioxanone implants: A systematic review on safety and performance in patients [file JBA888841_Supplemental_Material2.pdf]

Appendix 2 - Scoring system based on percentage of unfavourable outcomes in the analysed population in each publication.

<table>
<tr><th colspan="3">Outcomes</th><th>Score (%)</th></tr>
<tr><td rowspan="10">Unfavourable Outcomes (UO)</td><td rowspan="2">Surgical Site Infection (SSI)</td><td>SSI is commented or assessed</td><td>Patients with SSI</td></tr>
<tr><td>Not commented / not assessed</td><td>-</td></tr>
<tr><td rowspan="2">Inflammatory reaction</td><td>Inflammatory reaction is commented or assessed</td><td>Patients with inflammatory reaction</td></tr>
<tr><td>Not commented / not assessed</td><td>-</td></tr>
<tr><td rowspan="2">Foreign body reaction (FBR)</td><td>FBR is commented or assessed</td><td>Patients with FBR</td></tr>
<tr><td>Not commented / not assessed</td><td>-</td></tr>
<tr><td rowspan="2">Postoperative fever</td><td>Postoperative fever is commented or assessed</td><td>Patients with postoperative fever</td></tr>
<tr><td>Not commented / not assessed</td><td>-</td></tr>
<tr><td rowspan="2">Postoperative Pain</td><td>Postoperative pain is commented or assessed</td><td>Patients with postoperative pain</td></tr>
<tr><td>Not commented / not assessed</td><td>-</td></tr>
<tr><td colspan="2" rowspan="2">Performance</td><td>Performance is commented or assessed</td><td>Successful performance</td></tr>
<tr><td>Not commented / not assessed</td><td>-</td></tr>
</table>

Safety score (%) is calculated as 100 – (average of UO)
