# Supplementary material for: Polydioxanone implants: A systematic review on safety and performance in patients
Source: J Biomater Appl. 2019 Nov 26;34(7):902–16. doi: 10.1177/0885328219888841 (PMC7044756; doi:10.1177/0885328219888841)
Supplement: JBA888841 Supplemental Material3 - Supplemental material for Polydioxanone implants: A systematic review on safety and performance in patients [file JBA888841_Supplemental_Material3.pdf]

Appendix 3 - List of PDO implants approved by the FDA via 510(k) route.

510(k) Number	Type of Implant	Medical Device Brand Name	Company	Country	Approval Year	Regulation Medical Specialty
K812323	Clip	PDS* ABSORBABLE LIGATING CLIPS	Ethicon, Inc	USA	1981	General & Plastic Surgery
K844228	Mesh	PDS POLYDIOXANONE MESH	Ethicon, Inc.	USA	1985	General & Plastic Surgery
K864912	Pin	PDS (POLYDIOXANON) ABSORBABLE PIN	Johnson & Johnson Professionals, Inc.	USA	1987	Orthopaedic
K901456	Pin	ORTHOSORB® ABSORBABLE PIN	Johnson & Johnson International	USA	1990	Orthopaedic
K931492	Clip	LAPRA-TY* SUTURE CLIP	Ethicon, Inc.	USA	1993	General & Plastic Surgery
K970119	Staple	MITEK ABSORBABLE POLYDIOXANONE (PDS) H FIX	Mitek Products	USA	1998	Orthopaedic
K013274	Suture	MONO-DOX	Cp Medical	USA	2001	General & Plastic Surgery
K022666	Suture	PDO (POLYDIOXANONE) MONOFILAMENT SYNTHETIC ABSORBABLE SUTURE U.S.P.	Arc Medical Supplies (Beijing) Co, Ltd.	China	2002	General & Plastic Surgery
K030212	Suture	MONODEK	Genzyme Biosurgery	USA	2003	General & Plastic Surgery
K031216	Suture	MONOPLUS	Aesculap	USA	2003	General & Plastic Surgery
K031443	Suture	*SEW-RIGHT* Quick Load with 2-0 MONOGLIDE	Lsi Solutions	USA	2003	General & Plastic Surgery
K041048	Suture	ATRAMAT	Internacional Farmaceutica, S.A. De C.V.	Mexico	2004	General & Plastic Surgery

K042075	Suture	QUILL	Quill Medical, Inc.	USA	2004	General & Plastic Surgery
K042285	Suture	UNICRYL M	United Medical Industries Co. Ltd.	Kingdom of Saudi Arabia	2004	General & Plastic Surgery
K042517	Screw	BIOSORB FX AND BIOSORB PDX 1.5 AND 2.0 SCREWS	Linvatec Biomaterials, Ltd	Finland	2004	Dental
K051609	Suture	QUILL SYNTHETIC ABSORBABLE BARBED SUTURE	Quill Medical, Inc.	USA	2005	General & Plastic Surgery
K053380	Suture	CONTOUR THREAD SYNTHETIC ABSORBABLE PDO BARBED SUTURE	Surgical Specialties Corp.	USA	2006	General & Plastic Surgery
K061037	Suture	PDS PLUS ANTIBACTERIAL SUTURE	Ethicon, Inc.	USA	2006	General & Plastic Surgery
K063680	Suture	SHARPOINT PDO (POLYDIOXANONE) SUTURES	Surgical Specialties Corp.	USA	2007	General & Plastic Surgery
K071989	Suture	QUILL SELF-RETAINING SYSTEM (SRS) SYNTHETIC ABSORBABLE SURGICAL SUTURE MATERIAL	Surgical Specialties Corp.	USA	2007	General & Plastic Surgery
K080680	Suture	QUILL SELF-RETAINING SYSTEM (SRS) COMPRISED OF PDO	Surgical Specialties Corp.	USA	2008	General & Plastic Surgery
K080985	Suture	QUILL SELF-RETAINING SYSTEM (SRS) COMPRISED OF POLYDIOXANONE (PDO)	Surgical Specialties Corp.	USA	2008	General & Plastic Surgery
K081001	Suture	PD SYNTH	Sutures India Pvt., Ltd.	India	2008	General & Plastic Surgery
K082097	Suture	DemeTech POLYDIOXANONE SYNTHETIC MONOFILAMENT (PDO) ABSORBABLE SUTURE	Demetech Corp	USA	2010	General & Plastic Surgery
K092590	Plate	PDS Flexible Plates	Ethicon, Inc.	USA	2010	General & Plastic Surgery
K100461	Suture	PDM	Riverpoint Medical	USA	2010	General & Plastic Surgery
K100780	Suture	Polydioxanone Suture (Vital Sutures)	Unilene S.A.C.	USA	2010	General & Plastic Surgery
K113004	Suture	PDS™ BARBED SUTURES	Ethicon, Inc.	USA	2011	General & Plastic Surgery

K113744	Sutu re	QUILL PDO KNOTLESS TISSUE-CLOSURE DEVICE (POLYDIOXANONE)	Angiotech	USA	2012	General & Plastic Surgery
K120827	Sutu re	QUILL PDO KNOTLESS TISSUE-CLOSURE DEVICE	Angiotech	USA	2012	General & Plastic Surgery
K123877	Sutu re	QUILL PDO KNOTLESS TISSUE-CLOSURE DEVICE, VARIABLE LOOP DESIGN	Angiotech	USA	2013	General & Plastic Surgery
K130191	Sutu re	MINT (MINT Lift)	Hansbiomed Corporation	Korea	2013	General & Plastic Surgery
K132268	Sutu re	QUILL PDO KNOTLESS TISSUE-CLOSURE DEVICE	Angiotech	USA	2013	General & Plastic Surgery
K133420	Sutu re	TRANQUILL BARBED DEVICE	Angiotech	USA	2013	General & Plastic Surgery
K132669	Stap le	PROXIFAST ABSORBABLE STAPLE	Surgimatix, Inc.	USA	2014	General & Plastic Surgery
K141776	Sutu re	STRATAFIX	Ethicon, Inc.	USA	2014	General & Plastic Surgery
K143413	Sutu re	Y. Jacobs YOUNG'S Thread	Y. Jacobs Medical, Inc.	Korea	2015	General & Plastic Surgery
K150670	Sutu re	STRATAFIX Spiral PDS Plus Knotless Tissue Control Device	Ethicon, Inc.	USA	2015	General & Plastic Surgery
K150553	Sutu re	Filbloc	Assut Europe	Italy	2016	General & Plastic Surgery
K160705	Sutu re	Y.JACOBS YOUNG'S THREAD Synthetic Absorbable Surgical Fixation Suture	Y. Jacobs Medical, Inc.	USA	2016	General & Plastic Surgery
K160761	Sutu re	DW-1C	Dongwon Medical Co., Ltd.	Korea	2016	General & Plastic Surgery
K161737	Sutu re	RELI REDIDIOX Dyed, RELI REDIDIOX, RELI REDIDIOX Undyed	Myco Medical	USA	2017	General & Plastic Surgery
K172659	Sutu re	FILAXYN Absorbable Polydioxanone Surgical	Meril Endo Surgery Private Limited	India	2017	General & Plastic Surgery
K181094	Mes h	Polydioxanone Surgical Scaffold™	Surgical Innovation Associates, Inc	USA	2018	General & Plastic Surgery

K173917	Sutu re	neoClose PDS Anchor	neoSurgical Ltd.	Ireland	2018	Gastroenterology / Urology
K173874	Sutu re	Stitch Kit® Quill PDO	Origami Surgical LLC	USA	2018	General & Plastic Surgery
K182873	Sutu re	STRATAFIX™ Symmetric PDS™ Plus Knotless Tissue Control Device, STRATAFIX™ Spiral PDS™ Plus Knotless Tissue Control Device	Ethicon, Inc.	USA	2019	General & Plastic Surgery
