# Supplementary figures and images for: Polydioxanone implants: A systematic review on safety and performance in patients
Source: J Biomater Appl. 2019 Nov 26;34(7):902–16. doi: 10.1177/0885328219888841 (PMC7044756; doi:10.1177/0885328219888841)

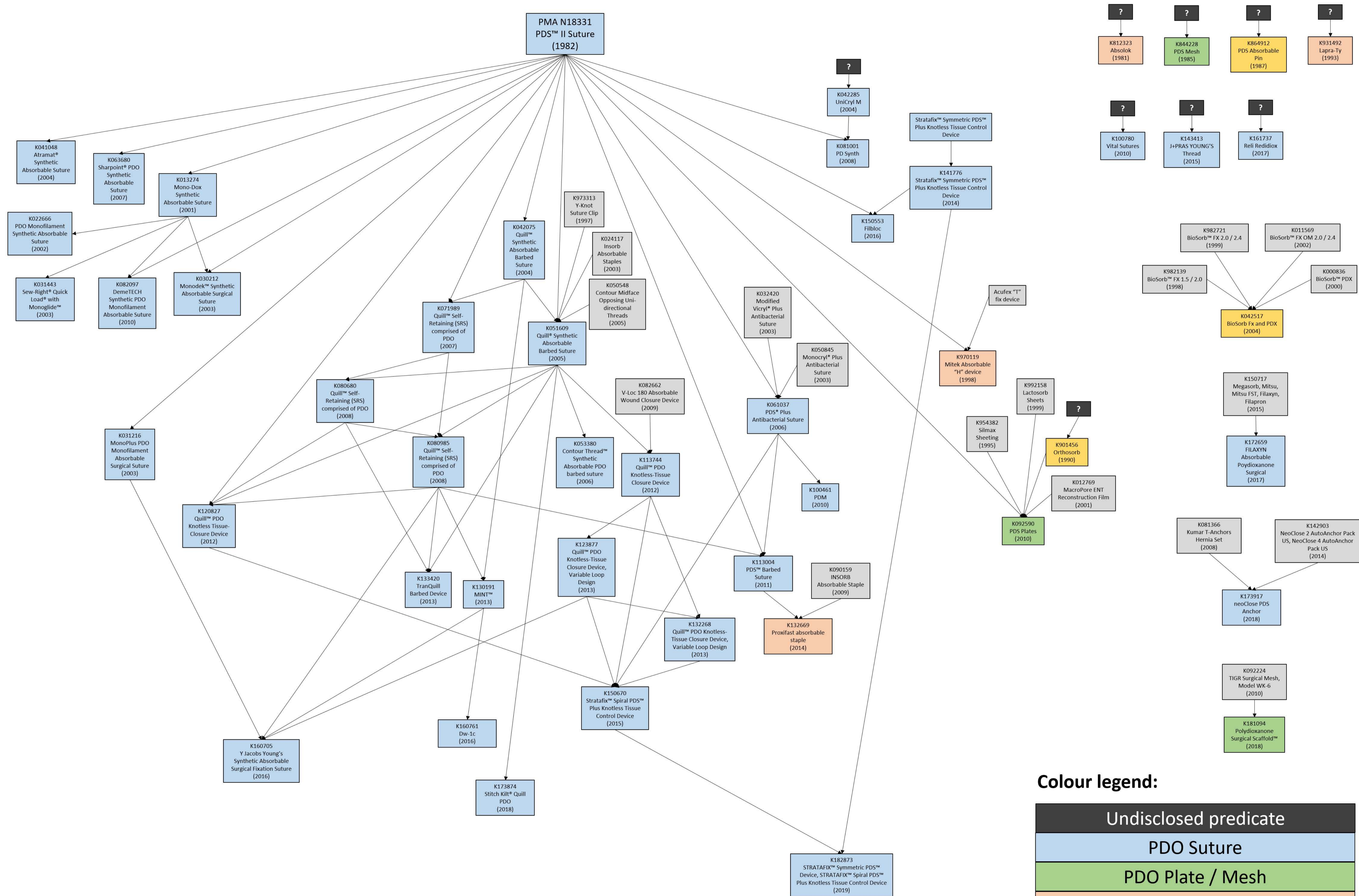

Supplement: JBA888841 Supplemental Material4 - Supplemental material for Polydioxanone implants: A systematic review on safety and performance in patients [file JBA888841_Supplemental_Material4.pdf]
