# Supplementary material for: Polydioxanone implants: A systematic review on safety and performance in patients
Source: J Biomater Appl. 2019 Nov 26;34(7):902–16. doi: 10.1177/0885328219888841 (PMC7044756; doi:10.1177/0885328219888841)
Supplement: JBA888841 Supplemental Material5 - Supplemental material for Polydioxanone implants: A systematic review on safety and performance in patients [file JBA888841_Supplemental_Material5.pdf]

Appendix 5 – Basic characteristics of the different types of PDO implants

PDO medical devices can be divided into four categories based on their shape: sutures, plates or meshes, screws or pins, and staples or clips.

Sutures can be categorised as monofilaments (only one strand) or multifilaments (more than one strand assembled together, either twisted or braided). Barbed sutures are a subcategory of monofilaments that have several barbs distributed over their length to provide better tissue attachment, while avoiding tissue retraction. When compared to smooth monofilament sutures of the same size, barbed sutures have a reduced tensile strength.[66] As an example, with regards to PDO medical devices, PDS™ II is considered as a monofilament suture, whereas Stratafix™ is considered a barbed suture. Both are manufactured by Ethicon Inc.

Plates or meshes are medical devices with a high surface area that provide mechanical support to tissues or organs during healing. Mesh fibre pattern, effective porosity and surface hydrophilicity are relevant to the biological tissue response and biocompatibility.[67] Scarring reactions/fibrosis can be found after incorporation of meshes into tissues, resulting from the surgical trauma during implantation or biological reaction post-surgery. Pore size should be considered when designing meshes as small pores are usually filled with inflammatory infiltrate or dense fibrotic scar, while large pores are filled with local physiological tissues once the mesh is implanted. Thus, large-pore meshes usually show less inflammation and fibrosis than small-pore meshes.[67] Mesh design should be suitable for the desired clinical application to avoid mesh-related adverse side effects and compromising clinical outcomes, by causing chronic pain, bacterial infection, restricted mobility or mesh migration, when implanted in a tissue with increased inflammation.[67] PDS™ Plate is a PDO plate manufactured by Ethicon Inc. This plate is commercialised as perforated or unperforated in different sizes and is mainly used in nasal reconstruction surgeries.

Polymer-based screws and pins are fixation devices that can overcome the issues seen with metallic screws including corrosion and patients with metal sensitivity. Additionally, when using absorbable screws or pins instead of metallic devices, the slow absorption process (more than six months) ensures secure fixation of the tissue throughout the healing process until it is no longer needed. An example of a PDO pin is OrthoSorb® (Johnson & Johnson International).

For a fast wound closure, clips or staples can be used either on their own or to secure sutures in place. Surgical staples are used when a good cosmetic closure is not a requirement. Typically, a surgeon inserts the two prongs of the staple through the tissue and then bend the staple prongs inward, grasping and securing the edges. Clips can be used to secure sutures in place by clamping it at the suture’s end, next to the tissue, preventing the suture from pulling through the tissue. Clips and staples usually have small dimensions when compared to the other medical device categories referred to here. Lapra-Ty® clips (Ethicon Inc.) are purple clips made of PDO.
