# Supplementary material for: Polydioxanone implants: A systematic review on safety and performance in patients
Source: J Biomater Appl. 2019 Nov 26;34(7):902–16. doi: 10.1177/0885328219888841 (PMC7044756; doi:10.1177/0885328219888841)
Supplement: JBA888841 Supplemental Material6 - Supplemental material for Polydioxanone implants: A systematic review on safety and performance in patients [file JBA888841_Supplemental_Material6.pdf]

Supplementary Data 1 - Safety and performance scores for PDO implants found in literature.

Outcomes	Monofilament/Multifilament PDO sutures												
	**Hehl et al.**[1]	**Murtha et al.**[2]	**Baracs et al.**[3]	**Justinger et al.**[4]	**Diener et al.**[5]	**Tan-Kim et al.**[6]	**Brolin**[7]	**Ulman et al.**[8]	**Seiler et al.**[9]	**Bloemen et al.**[10]	**Albertsmeier et al.**[11]	**Bassi and Tulandi**[12]	**Timmermans et al.**[13]
1. Surgical Site Infection	0.0	3.3	12.5	8.5	15.0	-	0.0	0.0	17.3	7.7	11.3	0.7	13.1
2. Inflammatory reaction	12.0	-	-	-	-	-	-	-	-	-	-	-	-
3. Foreign body reaction	-	19.7	-	-	-	-	-	-	-	-	-	-	-
4. Postoperative fever	-	-	-	-	-	-	-	-	-	-	-	0.0	-
5. Postoperative Pain	-	-	-	-	-	0.0	-	-	-	-	-	-	-
PDO device(s)	PDS™ II	PDS™ II	PDS™ Plus / PDS® II	PDS™ Plus / PDS® II	PDS™ Plus / PDS® II	PDS™ II / Monodek™	PDS™	PDS™	MonoPlus® / PDS™ II	PDS™	MonoPlus® / PDS®	PDS™	MonoPlus®
Number of patients	33	61	485	865	1185	32	120	61	415	233	141	139	107
Unfavourable Outcomes	6.0	11.5	12.5	8.5	15.0	0.0	0.0	0.0	17.3	7.7	11.3	0.4	13.1
**Safety Score**	**94.0**	**88.5**	**87.5**	**91.5**	**85.0**	**100.0**	**100.0**	**100.0**	**82.7**	**92.3**	**88.7**	**99.6**	**86.9**
**Performance Score**	**76.0**	**-**	**-**	**-**	**-**	**92.6**	**-**	**-**	**-**	**-**	**-**	**-**	**88.8**

**Outcomes**	**Monofilament/Multifilament PDO sutures**												
	**Kim-Fuchs et al.**[14]	**Allahdin, Glazener and Bain**[15]	**Bayraktar et al.**[16]	**Breuninger**[17]	**Cameron et al.**[18]	**Cassie et al.**[19]	**Chusak and Dibbell**[20]	**Constantine et al.**[21]	**Coras et al.**[22]	**Deerenberg et al.**[23]	**Fearon**[24]	**Gillatt et al.**[25]	**Varshney, Manek and Johnson**[26]
1. Surgical Site Infection	0.0	-	19.4	-	8.4	3.6	0.0	-	0.0	22.5	2.8	-	9.4
2. Inflammatory reaction	-	-		8.3	-	-	-	-	-	-	-	-	-
3. Foreign body reaction	-	-		-	-	-	-	-	-	-	-	-	-
4. Postoperative fever	-	-		-	-	-	-	-	-	-	-	-	-
5. Postoperative Pain	16.0	-		-	12.0	-	-	-	-	-	-	-	-
PDO device(s)	PDS™	PDS™	PDS™ II	PDS™	PDS™	PDS™	PDS™	PDS™	PDS™ II and Serasynth®	PDS™ Plus II	PDS™	PDS™	PDS™
Number of patients	133	33	101	665	143	28	52	2	26	545	137	46	100
Unfavourable Outcomes	8.0	-	19.4	8.3	10.2	3.6	0.0	-	0.0	22.5	2.8	-	9.4
**Safety Score**	**92.0**	**-**	**80.6**	**91.7**	**89.8**	**96.4**	**100.0**	**-**	**100.0**	**77.5**	**97.2**	**-**	**90.6**
**Performance Score**	**100.0**	**51.5**	**-**	**93.8**	**99.3**	**-**	**100.0**	**100.0**	**-**	**-**	**97.0**	**82.6**	**94.1**

Outcomes	Monofilament/Multifilament PDO sutures												
	Guyuron and Vaughan[27]	Helbling and Schlumpf[28]	Hohenleutner et al.[29]	Hoile[30]	Iwase et al.[31]	Justinger et al.[32]	Justinger et al.[33]	Kasturi et al.[34]	Khan et al.[35]	Kohler et al.[36]	Kreitmann, Riberi and Metras[37]	Leaper et al.[38]	Williams et al.[39]
1. Surgical Site Infection	0.0	4.2	0.0	8.5	6.6	10.8	9.2	-	-	26.1	0.0	17.0	12.0
2. Inflammatory reaction	-	-	-	-	-	-	-	-	-	-	0.0	-	-
3. Foreign body reaction	-	-	-	-	-	-	-	-	-	-	-	-	-
4. Postoperative fever	-	-	-	-	-	-	-	-	-	-	-	-	-
5. Postoperative Pain	-	29.2	-	-	-	-	-	-	-	44.1	-	-	-
PDO device(s)	Polydioxanone suture	PDS™	PDS™ II	PDS-C	PDS™ II	PDS™ II	PDS™ II	PDS™	PDS™	PDS™	PDS™	PDS™	PDS™
Number of patients	20	24	126	58	152	1045	409	65	1	81	50	107	100
Unfavourable Outcomes	0.0	16.7	0.0	8.5	6.6	10.8	9.2	-	-	35.1	0.0	17.0	12.0
**Safety Score**	**100.0**	**83.3**	**100.0**	**91.5**	**93.4**	**89.2**	**90.8**	**-**	**-**	**64.9**	**100.0**	**83.0**	**88.0**
**Performance Score**	**91.7**	**95.8**	**92.0**	**-**	**98.7**	**-**	**-**	**83.5**	**100.0**	**81.5**	**100.0**	**98.1**	**-**

Outcomes	Monofilament/Multifilament PDO sutures													
	**Luciani et al.**[40]	**Millbourn, Cengiz and Israelsson** [41]	**Muysoms et al.**[42]	**Nahas, Augusto and Ghelfond**[43]	**Nahas et al.**[44]	**Ohira et al.**[45]	**Okano et al.**[46]	**Parara et al.**[47]	**Ruiz-Tovar et al.**[48]	**Schenk, Landsiedl and Enenkel**[49]	**Spencker et al.** [50]	**Tan, Kannan and Page**[51]	**Yamaoka et al.** [52]	**Zhang et al.**[53]
1. Surgical Site Infection	-	7.8	0.0	-	-	7.4	-	0.0	24.5	-	1.1	-	3.1	0.8
2. Inflammatory reaction	-	-	-	-	-	-	-	-		-	-	-	-	-
3. Foreign body reaction	-	-	-	-	-	-	-	-		-	-	-	-	-
4. Postoperative fever	-	-	-	-	-	-	-	-		-	-	-	-	-
5. Postoperative Pain	-	-	2.0	-	-	-	-	-		-	-	-	-	-
PDO device(s)	PDS™	PDS™ II	PDS™	Polydioxanone Suture	PDS™	PDS™ II	PDS™	PDS™	PDS loop® and PDS Plus loop®	PDS-cord	Polydioxanone Suture	PDS™	PDS™ II	PDS™
Number of patients	181	737	59	10	12	27	11	40	53	13	185	7	323	118
Unfavourable Outcomes	-	7.8	1.0	-	-	7.4	-	0.0	24.5	-	1.1	-	3.1	0.8
**Safety Score**	**-**	**92.2**	**99.0**	**-**	**-**	**92.6**	**-**	**100.0**	**75.5**	**-**	**98.9**	**-**	**96.9**	**99.2**
**Performance Score**	**98.3**	**87.9**	**72.4**	**100.0**	**100.0**	**85.7**	**73.0**	**-**	**86.8**	**94.0**	**100.0**	**-**	**-**	**100.0**

Outcomes	Barbed PDO sutures											
	**Tan-Kim et al.[6]**	**Warner and Gutowski[54]**	**Murphey et al.[55]**	**Yeo, Lee and Han[56]**	**Murtha et al.[2]**	**Wright et al.[57]**	**Donnellan and Mansuria[58]**	**Kelley and Heller[59]**	**Liatsikos et al.[60]**	**Emanuelsson et al.[61]**	**Bogliolo et al.[62]**	**Giampaolino et al.[63]**
1. Surgical Site Infection	-	0.0	-	-	3.2	33.0	-	-	-	18.0	0.0	4.2
2. Inflammatory reaction	-	-	25.0	0.0	-	-	-	-	-	-	-	-
3. Foreign body reaction	-	-	-	-	10.2	-	-	-	-	-	-	-
4. Postoperative fever	-	-	-	-	-	0.0	0.0	-	-	-	4.2	8.3
5. Postoperative Pain	0.0	-	25.0	-	-	33.0	100.0	-	-	25.0	-	-
PDO device(s)	Quill™ SRS	Quill™	TranQuill	MINT Lift® / Omega 41®	Quill™ bidirectional barbed suture	Quill™ SRS	Quill™	Quil™ SRS	Quill™ SRS	Quill™ SRS	Quill™ SRS	Stratafix®
Number of patients	32	58	20	144	127	3	1	1	6	28	48	17
Unfavourable Outcomes	0.0	0.0	25.0	0.0	6.7	22.0	50.0	-	-	21.5	2.1	6.3
**Safety Score**	**100.0**	**100.0**	**75.0**	**100.0**	**93.3**	**78.0**	**50.0**	**-**	**-**	**78.5**	**97.9**	**93.8**
**Performance Score**	**89.3**	**-**	**45.0**	**97.2**	**-**	**0.0**	**0.0**	**100.0**	**16.7**	**96.4**	**-**	**82.4**

Outcomes	Barbed PDO sutures										
	Chan et al.[64]	Gililland et al.[65]	Blanc et al.[66]	Gys, Gys and Lafullarde[67]	Kang et al.[68]	Lee, Yoon and Lee[69]	Peleg et al.[70]	Shermak, Mallalieu and Chang[71]	Ting et al.[72]	Yanazume et al.[73]	Zayed et al.[74]
1. Surgical Site Infection	0.0	1.0	-	-	-	0.0	-	1.0	0.0	-	-
2. Inflammatory reaction	-	-	-	-	-	-	-	-	-	-	-
3. Foreign body reaction	-	-	-	-	0.0	-	-	-	-	-	-
4. Postoperative fever	-	-	-	-	-	-	-	-	-	-	2.0
5. Postoperative Pain	-	-	-	-	-	-	-	-	-	0.0	-
PDO device(s)	Stratafix®	Quill™ SRS	Stratafix®	Stratafix®	QTL LIFT™	PDO thread	Stratafix®	Quill™ SRS	Quill™	Stratafix®	Stratafix®
Number of patients	55	104	50	100	33	35	51	103	31	20	50
Unfavourable Outcomes	0.0	1.0	-	-	0.0	0.0	-	1.0	0.0	0.0	2.0
**Safety Score**	**100.0**	**99.0**	**-**	**-**	**100.0**	**100.0**	**-**	**99.0**	**100.0**	**100.0**	**98.0**
**Performance Score**	**96.4**	**-**	**100.0**	**60.0**	**84.8**	**100.0**	**68.6**	**17.5**	**-**	**-**	**96.0**

Outcomes	PDO Plates/Meshes										
	**Baumann et al.** [75]	**Rimmer, Ferguson and Saleh**[76]	**Fuller, Levesque and Lindsay**[77]	**Petropoulos et al.**[78]	**James and Kelly**[79]	**Boenisch and Trenité**[80]	**Tweedie, Lo and Rowe-Jones**[81]	**Dayan and Ashourian** [82]	**Becker et al.** [83]	**Daley et al.** [84]	**Dörfer et al.** [85]
1. Surgical Site Infection	-	2.0	2.6	-	1.7	0.0	-	6.7	0.0	-	6.7
2. Inflammatory reaction	3.2	-	-	0.0	-	0.0	-	-	-	-	-
3. Foreign body reaction	-	-	-	0.0	1.7	0.0	2.0	-	-	-	-
4. Postoperative fever	-	-	-	-	-	-	-	-	-	-	-
5. Postoperative Pain	-	-	2.6	-	-	-	-	-	-	-	-
PDO device(s)	PDO Sheets	Perforated PDO plate	Perforated and non perforated PDO plates	PDO foil	PDO foil	PDO plate	Unperforated or perforated PDO plate	PDO plate	PDS foil	Polydioxanone tape	Polydioxanone membrane (Mempol)
Number of patients	31	102	88	12	58	3	50	15	Not clear	45	15
Unfavourable Outcomes	3.2	2.0	2.6	0.0	1.7	0.0	2.0	6.7	0.0	-	6.7
**Safety Score**	**96.8**	**98.0**	**97.4**	**100.0**	**98.3**	**100.0**	**98.0**	**93.3**	**100.0**	**-**	**93.3**
**Performance Score**	**67.0**	**95.1**	**79.5**	**90.9**	**100.0**	**87.5**	**86.0**	**100.0**	**-**	**78.0**	**-**

Outcomes	PDO Plates/Meshes										
	**Eickholz et al.** [86]	**Eickholz et al.** [87]	**Epprecht et al.** [88]	**Gierloff et al.**[89]	**Iizuka et al.**[90]	**Kontio et al.**[91]	**Krokidis et al.**[92]	**Moina, Moina and Racanti** [93]	**Pau et al.** [94]	**Repici et al.** [95]	**Sand, Desai and Branham**[96]
1. Surgical Site Infection	4.5	3.8	5.0	0.0	0.0	-	-	-	0.0	-	-
2. Inflammatory reaction	-	-	-	-	0.0	6.3	18.2	-	-	-	-
3. Foreign body reaction	-	-	-	-	-	-	-	-	-	-	-
4. Postoperative fever	-	-	-	-	-	-	-	-	-	-	-
5. Postoperative Pain	-	-	5.0	-	-	-	-	-	-	-	-
PDO device(s)	Polydioxanone membrane (Mempol)	Polydioxanone membrane (Mempol)	PDS plate	PDS foil	PDS Plate	PDS plate or sheet	Oesophageal Degradable BD SX-ELLA Stent	PDS plate	PDS sheet	Ella Stent	Polydioxanone plate
Number of patients	21	13	20	194	20	16	11	10	19	11	7
Unfavourable Outcomes	4.5	3.8	5.0	0.0	0.0	6.3	18.2	-	0.0	-	-
**Safety Score**	**95.5**	**96.2**	**95.0**	**100.0**	**100.0**	**93.8**	**81.8**	**-**	**100.0**	**-**	**-**
**Performance Score**	**-**	**-**	**95.0**	**75.0**	**92.6**	**-**	**18.2**	**100.0**	**94.7**	**45.5**	**85.7**

Outcomes	PDO Screws/Pins						PDO Clips/Staples			
	Small, Braly and Tullos[97]	Kalla and Janzen[98]	Chandran, Kamath and Nihal[99]	Prior et al.[100]	Gill et al.[101]	McManners, Moos and El-Attar[102]	Finley et al.[103]	Miller et al.[104]	Brusky and Tran[105]	Yasui et al.[106]
1. Surgical Site Infection	0.0	0.0	-	0.0	1.7	-	100.0	-	100.0	-
2. Inflammatory reaction	-	50.0	-	-	-	-	-	-	-	-
3. Foreign body reaction	-	50.0	-	0.0	-	-	-	-	-	-
4. Postoperative fever	-	-	-	-	-	-	-	-	100.0	-
5. Postoperative Pain	-	50.0	0.0	0.0	-	-	-	100.0	100.0	-
PDO device(s)	Orthosorb®	Orthosorb®	Orthosorb®	Orthosorb®	Orthosorb®	Orthosorb®	Lapra-Ty® suture clips	Lapra-Ty® suture clips	Lapra-Ty® suture clips	Lapra-Ty® suture clips
Number of patients	71	1	1	39	144	10	1	1	1	30
Unfavourable Outcomes	0.0	37.5	0.0	0.0	1.7	-	100.0	100.0	100.0	-
**Safety Score**	**100.0**	**62.5**	**100.0**	**100.0**	**98.3**	**-**	**0.0**	**0.0**	**0.0**	**-**
**Performance Score**	**84.0**	**50.0**	**100.0**	**95.2**	**-**	**78.0**	**0.0**	**0.0**	**0.0**	**90.0**

Supplementary Data 2 - Safety and performance scores for PDO implants found in literature, when compared to non PDO alternatives.

Outcomes	PDO suture vs non-PDO sutures											
	Brolin[7]	Ulman et al. [8]	Seiler et al.[9]	Bloemen et al.[10]	Albertsmeier et al.[11]	Gililland et al.[65]	Neubauer et al.[107]	Bassi and Tulandi[12]	Bogliolo et al.[62]	Giampaolino et al.[63]	Chan et al.[64]	Allahdin, Glazener and Bain[15]
1. Surgical Site Infection	2	2	0	0	0	0	-	0	-	0	2	-
2. Inflammatory reaction	-	-	-	-	-	-	-	-	-	-	-	-
3. Foreign body reaction	-	-	-	-	-	-	-	-	-	-	-	-
4. Postoperative fever	-	-	-	-	-	-	-	2	0	0	-	-
5. Postoperative Pain	-	-	-	-	-	-	-	-	-	0	-	-
PDO Device(s)	PDS™	PDS™	MonoPlus® / PDS™ II	PDS™	MonoPlus® / PDS®	Quill™ SRS PDO	Monofilament polydioxanone suture	PDS™	Quill™ SRS	Stratafix®	Stratafix®	PDS™
Non-PDO alternative(s)	Ethibond	VICRYL®	VICRYL®	Prolene®	MonoMax®	Ethibond™ / MONOCRYL®	V-Loc 180	V-Loc	VICRYL®	VICRYL®	VICRYL®	VICRYL®
Number of patients in PDO group	120	61	415	233	141	98	58	139	48	17	55	33
**Safety Score**	**2**	**2**	**0**	**0**	**0**	**0**	**-**	**1**	**0**	**0**	**2**	**-**
**Performance Score**	**2**	**2**	**-**	**-**	**-**	**0**	**-**	**-**	**-**	**2**	**2**	**0**

Outcomes	PDO suture vs non-PDO sutures											
	**Breuninger**[17]	**Cameron et al.**[18]	**Cassie et al.**[19]	**Chusak and Dibell**[20]	**Ganesh et al.**[108]	**Gillatt et al.**[25]	**Gupta et al.**[109]	**Guyuron and Vaughan**[27]	**Gys, Gys and Lafullarde**[110]	**Hohenleutner et al.**[29]	**Iwase et al.**[31]	**Justinger et al.**[32]
1. Surgical Site Infection	-	0	1	2	-	-	-	2	-	2	0	-2
2. Inflammatory reaction	0	-	-	-	-	-	-	-	-	-	-	-
3. Foreign body reaction	-	-	-	-	-	-	-	-	-	-	-	-
4. Postoperative fever	-	-	-	-	-	-	-	-	-	-	-	-
5. Postoperative Pain	-	0	1	-	-	-	-	-	-	-	-	-
PDO Device(s)	PDS™	PDS™	PDS™	PDS™	Quill™ PDO	PDS™	PDS®	PDS™	Stratafix®	PDS™ II	PDS™ II	PDS™ II
Non-PDO alternative(s)	Mason 10® and Vicryl and Monocryl®	Prolene®	Nylon	Catgut suture	VICRYL®	Silk or chromic catgut	VICRYL®	Polyglactin 910 Suture	VICRYL®	VICRYL®	Braided Silk	VICRYL® Plus
Number of patients in PDO group	665	143	28	52	2	46	30	12	100	126	152	1045
**Safety Score**	**0**	**0**	**1**	**2**	**-**	**-**	**-**	**2**	**-**	**2**	**0**	**-2**
**Performance Score**	**0**	**2**	**-**	**2**	**-**	**0**	**2**	**2**	**2**	**2**	**2**	**-**

Outcomes	PDO suture vs non-PDO sutures												
	Justinger et al.[33]	Kohler et al.[36]	Leaper et al.[38]	Luciani et al.[40]	Nahas, Augusto and Ghelfond[43]	Ohira et al.[45]	Parara et al.[47]	Peleg et al.[70]	Ruiz-Tovar et al.[48]	Zhang et al.[53]	Ting et al.[72]	Yanazume et al.[73]	Zayed et al.[74]
1. Surgical Site Infection	-2	0	0	-	-	0	2	-	-	0	2	-	-
2. Inflammatory reaction	-	-	-	-	-	-	-	-	-	-	-	-	-
3. Foreign body reaction	-	-	-	-	-	-	-	-	-	-	-	-	-
4. Postoperative fever	-	-	-	-	-	-	-	-	-	-	-	-	0
5. Postoperative Pain	-	1	-	-	-	-	-	-	-	-	-	2	-
PDO Device(s)	PDS™ II	PDS™	PDS™	PDS™	Polydioxanone	PDS™ II	PDS II™	Stratafix®	PDS loop® and PDS Plus loop®	PDS™	Quill™	Stratafix®	Stratafix®
Non-PDO alternative(s)	VICRYL® Plus	Dynamesh-IPOM	Nylon	Stainless Steel Wire	Mononylon	Polysorb	Polypropylene and Ethilon and APPOSE and Vicryl Rapide	VICRYL® Plus	Vicryl loop® and Vicryl Plus loop®	Silk Thread	VICRYL®	VICRYL®	VICRYL®
Number of patients in PDO group	409	81	107	181	10	27	40	51	53	118	31	20	50
**Safety Score**	**-2**	**0,5**	**0**	**-**	**-**	**0**	**2**	**-**	**-**	**0**	**2**	**2**	**0**
**Performance Score**	**-**	**-1**	**0**	**2**	**0**	**0**	**2**	**2**	**-1**	**0**	**0**	**-**	**2**

Outcomes	PDO suture vs other non-PDO devices										
	**Obwegeser[111]**	**Emanuelsson et al.[61]**	**Kim-Fuchs et al.[14]**	**Helbling and Schlumpf[28]**	**Muysoms et al.[42]**	**Okano et al.[46]**	**Schenk, Landsiedl and Enenkel[49]**	**Spencker et al.[50]**	**Timmermans et al.[13]**	**Yamaoka et al.[52]**	**Shermak, Mallalieu and Chang[71]**
1. Surgical Site Infection	-	0	2	0	2	-	-	0	0	1	1
2. Inflammatory reaction	-	-	-	-	-	-	-	-	-	-	-
3. Foreign body reaction	-	-	-	-	-	-	-	-	-	-	-
4. Postoperative fever	-	-	-	-	-	-	-	-	-	-	-
5. Postoperative Pain	-	0	0	-	0	-	-	-	-	-	-
PDO Device(s)	PDS™ II	Quill™ SRS	PDS™	PDS™	PDS™	PDS™	PDS-cord	Polydioxanone suture	MonoPlus	PDS™ II	Quill™ SRS
Non-PDO alternative(s)	Titanium miniplates	BARD™ Soft Mesh	Histoacryl	Histoacryl	Polypropylene mesh	Stapling device	Tendon augmentation	2-octyl-cyanoacrylate adhesive	Polypropylene mesh	3M™ Skin Stapler	No barbed suture
Number of patients in PDO group	15	28	133	24	59	11	13	185	107	323	103
**Safety Score**	**-**	**0**	**1**	**0**	**1**	**-**	**-**	**0**	**0**	**1**	**1**
**Performance Score**	**0**	**0**	**0**	**0**	**-1**	**-1**	**0**	**2**	**0**	**-**	**0**

Outcomes	PDO Plates/Meshes vs non-PDO devices							PDO Screws vs non-PDO devices		PDO Clips vs no clips
	Dayan and Ashourian[82]	Becker et al.[83]	Christgau et al.[112]	Dörfer et al.[85]	Eickholz et al.[86]	Eickholz et al.[87]	Pretzl et al.[113]	Prior et al.[100]	Gill et al.[101]	Yasui[106]
1. Surgical Site Infection	0	2	-	0	-	-	-	2	0	-
2. Inflammatory reaction	-	-	-	-	-	-	-	-	-	-
3. Foreign body reaction	-	-	-	-	-	-	-	-	-	-
4. Postoperative fever	-	-	-	-	-	-	-	-	-	-
5. Postoperative Pain	-	-	-	-	-	-	-	2	-	-
PDO Device(s)	PDS™ plate	PDS foil	PDS membrane (Mempol)	Polydioxanone membrane (Mempol)	Polydioxanone membrane (Mempol)	Polydioxanone membrane (Mempol)	Polydioxanone membrane (Mempol)	Orthosorb®	Orthosorb®	Lapra-Ty® suture clips
Non-PDO alternative(s)	Graft without PDS plate	Collagen membrane	PLA membrane	Polylactide acetyltributyl citrate membrane (Guidor)	Polylactide acetyltributyl citrate membrane (Guidor Matrix Barrier)	Polylactide acetyltributyl citrate membrane (Guidor Matrix Barrier)	Polylactide acetyltributyl citrate membrane (Guidor Matrix Barrier)	K wires / VICRYL®	Kirschner wires	No clips
Number of patients in PDO group	15	Not clear	62	15	21	13	13	39	144	30
**Safety Score**	**0**	**2**	**-**	**0**	**-**	**-**	**-**	**2**	**0**	**-**
**Performance Score**	**0**	**0**	**0**	**0**	**0**	**2**	**0**	**0**	**0**	**2**

References:

1. Hehl G, Strecker W, Richter M, et al. Clinical experience with PDS II augmentation for operative treatment of acute proximal ACL ruptures - 2-year follow-up. *Knee Surg Sports Traumatol Arthrosc* 1999; 7: 102–106.
2. Murtha AP, Kaplan AL, Paglia MJ, et al. Evaluation of a Novel Technique for Wound Closure Using a Barbed Suture. *Plast Reconstr Surg* 2006; 117: 1769–1780.
3. Baracs J, Huszár O, Sajjadi SG, et al. Surgical Site Infections after Abdominal Closure in Colorectal Surgery Using Triclosan-Coated Absorbable Suture (PDS Plus) vs. Uncoated Sutures (PDS II): A Randomized Multicenter Study. *Surg Infect (Larchmt)* 2011; 12: 483–489.
4. Justinger C, Slotta JE, Ningel S, et al. Surgical-site infection after abdominal wall closure with triclosan-impregnated polydioxanone sutures: Results of a randomized clinical pathway facilitated trial (NCT00998907). *Surg (United States)* 2013; 154: 589–595.
5. Diener MK, Knebel P, Kieser M, et al. Effectiveness of triclosan-coated PDS Plus versus uncoated PDS II sutures for prevention of surgical site infection after abdominal wall closure: The randomised controlled PROUD trial. *Lancet* 2014; 384: 142–152.
6. Tan-Kim J, Nager CW, Grimes CL, et al. A randomized trial of vaginal mesh attachment techniques for minimally invasive sacrocolpopexy. *Int Urogynecol J Pelvic Floor Dysfunct* 2015; 26: 649–656.
7. Brolin RE. Prospective, randomized evaluation of midline fascial closure in gastric bariatric operations. *Am J Surg* 1996; 172: 328–331.
8. Ulman I, Erikçi V, Avanoğlu A, et al. The effect of suturing technique and material on complication rate following hypospadias repair. *Eur J Pediatr Surg* 1997; 7: 156–7.
9. Seiler CM, Bruckner T, Diener MK, et al. Interrupted or Continuous Slowly Absorbable Sutures For Closure of Primary Elective Midline Abdominal Incisions. *Ann Surg* 2009; 249: 576–582.
10. Bloemen A, Van Dooren P, Huizinga BF, et al. Randomized clinical trial comparing polypropylene or polydioxanone for midline abdominal wall closure. *Br J Surg* 2011; 98: 633–639.
11. Albertsmeier M, Seiler CM, Fischer L, et al. Evaluation of the safety and efficacy of MonoMax® suture material for abdominal wall closure after primary midline laparotomy - A controlled prospective multicentre trial: ISSAAC [NCT005725079]. *Langenbeck's Arch Surg* 2012; 397: 363–371.
12. Bassi A, Tulandi T. Evaluation of Total Laparoscopic Hysterectomy With and Without the Use of Barbed Suture. *J Obstet Gynaecol Canada* 2013; 35: 718–722.
13. Timmermans L, Eker HH, Steyerberg ES, et al. Short-term results of a randomized controlled trial comparing primary suture with primary glued mesh augmentation to prevent incisional hernia. *Ann Surg* 2015; 261: 276–281.
14. Kim-Fuchs C, Angst E, Vorburger S, et al. Prospective randomized trial comparing sutured with sutureless mesh fixation for Lichtenstein hernia repair: long-term results. *Hernia* 2012; 16: 21–27.
15. Allahdin S, Glazener C, Bain C. A randomised controlled trial evaluating the use of polyglactin mesh, polydioxanone and polyglactin sutures for pelvic organ prolapse surgery. *J Obstet Gynaecol (Lahore)* 2008; 28: 427–431.

16. Bayraktar B, Özemir IA, Saɫiroɫlu J, et al. A retrospective analysis of early and late term complications in patients who underwent application of retention sutures for gastrointestinal tract malignancies. *Turkish J Surg* 2015; 31: 15–19.
17. Breuninger H. Intracutaneous butterfly suture - A horizontal buried interrupted suture for high tension. *Eur J Plast Surg* 1998; 21: 415–419.
18. Cameron AEP, Parker CJ, Field ES, et al. A randomised comparison of polydioxanone (PDS®) and polypropylene (Prolene®) for abdominal wound closure. *Ann R Coll Surg Engl* 1987; 69: 113–115.
19. Cassie AB, Chatterjee AK, Mehta S, et al. Pain quantum and wound healing: A comparison of interrupted inversion PDS and standard nylon sutures in abdominal skin closure. *Ann R Coll Surg Engl* 1988; 70: 339–342.
20. Chusak RB, Dibbell DG. Clinical Experience with Polydioxanone Monofilament Absorbable Sutures in Plastic Surgery. *Plast Reconstr Surg* 1983; 72: 217–220.
21. Constantine FC, Ahmad J, Geissler P, et al. Simplifying the management of caudal septal deviation in rhinoplasty. *Plast Reconstr Surg* 2014; 134: 379e-388e.
22. Coras B, Hohenleutner U, Landthaler M, et al. Comparison of two absorbable monofilament polydioxanone threads in intradermal buried sutures. *Dermatologic Surg* 2005; 31: 331–333.
23. Deerenberg EB, Harlaar JJ, Steyerberg EW, et al. Small bites versus large bites for closure of abdominal midline incisions (STITCH): A double-blind, multicentre, randomised controlled trial. *Lancet* 2015; 386: 1254–1260.
24. Fearon JA. Rigid fixation of the calvaria in craniosynostosis without using 'rigid' fixation. *Plastic and Reconstructive Surgery* 2003; 111: 27–38.
25. Gillatt DA, Corfield AP, May RE, et al. Polydioxanone suture in the gastrointestinal tract. *Ann R Coll Surg Engl* 1987; 69: 54–56.
26. Varshney S, Manek P, Johnson C. Six-fold suture: wound length ratio for abdominal closure. *Ann R Coll Surg Engl* 1999; 81: 333–336.
27. Guyuron B, Vaughn C. Comparison of Polydioxanone and Polyglactin 910 in Intradermal Repair. *Plast Reconstr Surg* 1986; 98: 817–820.
28. Helbling C, Schlumpf R. Sutureless Lichtenstein: First results of a prospective randomised clinical trial. *Hernia* 2003; 7: 80–84.
29. Hohenleutner U, Egner N, Hohenleutner S, et al. Intradermal buried vertical mattress suture as sole skin closure: Evaluation of 149 cases. *Acta Derm Venereol* 2000; 80: 344–347.
30. Hoile RW. The use of a new suture material (polydioxanone) in the biliary tract. *Ann R Coll Surg Engl* 1983; 65: 168–171.
31. Iwase K, Higaki J, Tanaka Y, et al. Running closure of clean and contaminated abdominal wounds using a synthetic monofilament absorbable looped suture. *Surg Today* 1999; 29: 874–879.
32. Justinger C, Moussavian MR, Schlueter C, et al. Antibiotic coating of abdominal closure sutures and wound infection. *Surgery* 2009; 145: 330–334.
33. Justinger C, Schuld J, Sperling J, et al. Triclosan-coated sutures reduce wound infections after hepatobiliary surgery-a prospective non-randomized clinical pathway driven study. *Langenbeck's Arch Surg* 2011; 396: 845–850.
34. Kasturi S, Bentley-Taylor M, Woodman PJ, et al. High uterosacral ligament vaginal vault suspension: Comparison of absorbable vs. permanent

suture for apical fixation. *Int Urogynecol J* 2012; 23: 941–945.
35. Abbas Khan MA, Bhutto AM, Farid M, et al. Aesthetic sculpting for the natal cleft and buttock lift using an innovative periosteal-dermal suture suspension technique. *J Craniofac Surg* 2012; 23: 172–174.
36. Kohler A, Lavanchy JL, Lenoir U, et al. Effectiveness of Prophylactic Intraperitoneal Mesh Implantation for Prevention of Incisional Hernia in Patients Undergoing Open Abdominal Surgery: A Randomized Clinical Trial. *JAMA Surg* 2019; 154: 150–158.
37. Kreitmann B, Riberi A, Metras D. Evaluation of an Absorbable Suture for Sternal Closure in Pediatric Cardiac Surgery. *J Card Surg* 1992; 7: 254–256.
38. Leaper DJ, Allan A, May RE, et al. Abdominal wound closure: A controlled trial of polyamide (Nylon) and polydioxanone suture (PDS). *Ann R Coll Surg Engl* 1985; 67: 273–275.
39. Williams ZF, Tenzel P, Hooks WB, et al. Suture to wound length ratio in abdominal wall closure: how well are we doing? *Hernia* 2017; 21: 869–872.
40. Luciani N, Anselmi A, Gandolfo F, et al. Polydioxanone sternal sutures for prevention of sternal dehiscence. *J Card Surg* 2006; 21: 580–584.
41. Millbourn D, MD; Cengiz Y, MD, PhD; Israelsson L A., MD P. Effect of Stitch Length on Wound Complications After Closure of Midline Incisions. *Arch Surg* 2009; 144: 1056–1059.
42. Muysoms FE, Detry O, Vierendeels T, et al. Prevention of Incisional Hernias by Prophylactic Mesh-augmented Reinforcement of Midline Laparotomies for Abdominal Aortic Aneurysm Treatment. *Ann Surg* 2016; 263: 638–645.
43. Nahas FX, Augusto SM, Ghelfond C. Nylon Versus Polydioxanone in the Correction of Rectus Diastasis.pdf. *Plast Reconstr Surg* 2001; 107: 700–706.
44. Nahas FX, Ferreira LM, Ely PB, et al. Rectus diastasis corrected with absorbable suture: A long-term evaluation. *Aesthetic Plast Surg* 2011; 35: 43–48.
45. Ohira G, Kawahira H, Miyauchi H, et al. Synthetic polyglycomer short-term absorbable sutures vs. polydioxanone long-term absorbable sutures for preventing incisional hernia and wound dehiscence after abdominal wall closure: a comparative randomized study of patients treated for gastric or colon. *Surg Today* 2015; 45: 841–845.
46. Okano K, Kakinoki K, Yachida S, et al. A simple and safe pancreas transection using a stapling device for a distal pancreatectomy. *J Hepatobiliary Pancreat Surg* 2008; 15: 353–358.
47. Parara SM, Manios A, De Bree E, et al. Significant differences in skin irritation by common suture materials assessed by a comparative computerized objective method. *Plast Reconstr Surg* 2011; 127: 1191–1198.
48. Ruiz-Tovar J, Alonso N, Ochagavía A, et al. Effect of the Abdominal Fascial Closure with Triclosan-Coated Sutures in Fecal Peritonitis, on Surgical Site Infection, and Evisceration: A Retrospective Multi-Center Study. *Surg Infect (Larchmt)* 2018; 19: 61–64.
49. Schenk S, Landsiedl F, Enenkel M. Arthroscopic single-stranded semitendinosus tendon- versus PDS-augmentation of reinserted acute femoral

anterior cruciate ligament tears: 7 year follow-up study. *Knee Surgery, Sport Traumatol Arthrosc* 2006; 14: 318–324.
50. Spencker S, Coban N, Koch L, et al. Comparison of skin adhesive and absorbable intracutaneous suture for the implantation of cardiac rhythm devices. *Europace* 2011; 13: 416–420.
51. Tan EK, Kannan RY, Page RE. The use of Vicryl^{TM} in extensor tendon repairs. *Eur J Plast Surg* 2009; 32: 19–22.
52. Yamaoka Y, Ikeda M, Ikenaga M, et al. Efficacy of skin closure with subcuticular sutures for preventing wound infection after resection of colorectal cancer: a propensity score–matched analysis. *Langenbeck's Arch Surg* 2015; 400: 961–966.
53. Zhang J, Zhang HK, Zhu HY, et al. Mass Continuous Suture versus Layered Interrupted Suture in Transverse Abdominal Incision Closure after Liver Resection. *World J Surg* 2016; 40: 2237–2244.
54. Warner JP, Gutowski KA. Abdominoplasty With Progressive Tension Closure Using A Barbed Suture Technique. *Aesthetic Surg J* 2009; 29: 221–225.
55. Murphey AW, Nguyen SA, Fuller C, et al. TranQuill sling snoreplasty for snoring: A single-arm pilot study for safety and effectiveness. *Laryngoscope* 2016; 126: 243–248.
56. Yeo SH, Lee YB, Han DG, et al. Early Complications from Absorbable Anchoring Suture Following Thread-Lift for Facial Rejuvenation. *Arch Aesthetic Plast Surg* 2017; 23: 11–16.
57. Wright RC, Gillis CT, Yacoubian S V., et al. Extensor mechanism repair failure with use of bidirectional barbed suture in total knee arthroplasty. *J Arthroplasty* 2012; 27: 1–4.
58. Donnellan NM, Mansuria SM. Small Bowel Obstruction Resulting from Laparoscopic Vaginal Cuff Closure with a Barbed Suture. *J Minim Invasive Gynecol* 2011; 18: 528–530.
59. Kelley BP, Heller L. A novel approach to repair of wound dehiscence in the complicated patient. *Hernia* 2012; 16: 369–372.
60. Liatsikos E, Knoll T, Kyriazis I, et al. Unfavorable outcomes of laparoscopic pyeloplasty using barbed sutures: A multi-center experience. *World J Urol* 2013; 31: 1441–1444.
61. Emanuelsson P, Gunnarsson U, Strigård K, et al. Early complications, pain, and quality of life after reconstructive surgery for abdominal rectus muscle diastasis: A 3-month follow-up. *J Plast Reconstr Aesthetic Surg* 2014; 67: 1082–1088.
62. Bogliolo S, Nadalini C, Iacobone AD, et al. Vaginal cuff closure with absorbable bidirectional barbed suture during total laparoscopic hysterectomy. *Eur J Obstet Gynecol Reprod Biol* 2013; 170: 219–221.
63. Giampaolino P, De Rosa N, Tommaselli GA, et al. Comparison of bidirectional barbed suture Stratafix and conventional suture with intracorporeal knots in laparoscopic myomectomy by office transvaginal hydrolaparoscopic follow-up: A preliminary report. *Eur J Obstet Gynecol Reprod Biol* 2015; 195: 146–150.
64. Chan VWK, Chan P-K, Chiu K-Y, et al. Does Barbed Suture Lower Cost and Improve Outcome in Total Knee Arthroplasty? A Randomized Controlled Trial. *J Arthroplasty* 2017; 32: 1474–1477.

65. Gililland JM, Anderson LA, Sun G, et al. Perioperative closure-related complication rates and cost analysis of barbed suture for closure in TKA. *Clin Orthop Relat Res* 2012; 470: 125–129.
66. Blanc P, Lointier P, Breton C, et al. The Hand-sewn Anastomosis with an Absorbable Bidirectional Monofilament Barbed Suture Stratafix® During Laparoscopic One Anastomosis Loop Gastric Bypass. Retrospective Study in 50 Patients. *Obes Surg* 2015; 25: 2457–2460.
67. Gys B, Gys T, Lafullarde T. The Use of Unidirectional Knotless Barbed Suture for Enterotomy Closure in Roux-en-Y Gastric Bypass: a Randomized Comparative Study. *Obes Surg* 2017; 27: 2159–2163.
68. Kang SH, Moon SH, Rho B Il, et al. Wedge-shaped polydioxanone threads in a folded configuration (“Solid fillers”): A treatment option for deep static wrinkles on the upper face. *J Cosmet Dermatol* 2019; 18: 65–70.
69. Lee H, Yoon K, Lee M. Outcome of facial rejuvenation with polydioxanone thread for Asians. *J Cosmet Laser Ther* 2018; 20: 189–192.
70. Peleg D, Ahmad RS, Warsof SL, et al. A randomized clinical trial of knotless barbed suture vs conventional suture for closure of the uterine incision at cesarean delivery. *Am J Obstet Gynecol* 2018; 218: 343.e1-343.e7.
71. Shermak MA, Mallalieu J, Chang D. Barbed suture impact on wound closure in body contouring surgery. *Plast Reconstr Surg* 2010; 126: 1735–1741.
72. Ting NT, Moric MM, Della Valle CJ, et al. Use of Knotless Suture for Closure of Total Hip and Knee Arthroplasties. A Prospective, Randomized Clinical Trial. *J Arthroplasty* 2012; 27: 1783–1788.
73. Yanazume S, Togami S, Fukuda M, et al. New Continuous Barbed Suture Device with Stratafix for the Vaginal Stump in Laparoscopic Hysterectomy. *Gynecol Minim Invasive Ther* 2018; 7: 167–171.
74. Zayed MA, Fouda UM, Elsetohy KA, et al. Barbed sutures versus conventional sutures for uterine closure at cesarean section; a randomized controlled trial. *J Matern Neonatal Med* 2019; 32: 710–717.
75. Baumann A, Burggasser G, Gauss N, et al. Orbital floor reconstruction with an alloplastic resorbable polydioxanone sheet. *Int J Oral Maxillofac Surg* 2002; 31: 367–373.
76. Rimmer J, Ferguson LM, Saleh HA. Versatile applications of the polydioxanone plate in rhinoplasty and septal surgery. *Arch Facial Plast Surg* 2013; 14: 323–30.
77. Fuller JC, Levesque PA, Lindsay RW. Polydioxanone plates are safe and effective for L-strut support in functional septorhinoplasty. *Laryngoscope* 2017; 00: 8–10.
78. Petropoulos I, Nolst Trenite G, Boenisch M, et al. External septal reconstruction with the use of polydioxanone foil: Our experience. *Eur Arch Oto-Rhino-Laryngology* 2006; 263: 1105–1108.
79. James SE, Kelly MH. Cartilage recycling in rhinoplasty: polydioxanone foil as an absorbable biomechanical scaffold. *Plast Reconstr Surg* 2008; 122: 254–60.
80. Huang K, Ding X, Lv B, et al. Reconstruction of large-size abdominal wall defect using biodegradable poly-p-dioxanone mesh: An experimental

canine study. *World J Surg Oncol* 2014; 12: 1–8.
81. Tweedie DJ, Lo S, Rowe-Jones JM. Reconstruction of the nasal septum using perforated and unperforated polydioxanone foil. *Arch Facial Plast Surg* 2010; 12: 106–113.
82. Dayan SH, Ashourian N. Polydioxanone Absorbable Plate for Cartilaginous Grafting in Endonasal Rhinoplasty. *JAMA Facial Plast Surg* 2016; 18: 47–53.
83. Becker ST, Terheyden H, Fabel M, et al. Comparison of collagen membranes and polydioxanone for reconstruction of the orbital floor after fractures. *J Craniofac Surg* 2010; 21: 1066–1068.
84. Daley M, Brizard CP, Konstantinov IE, et al. Absorbable pulmonary artery banding: A strategy for reducing reoperations. *Eur J Cardio-thoracic Surg* 2017; 51: 735–739.
85. Dörfer CE, Kim TS, Steinbrenner H, et al. Regenerative periodontal surgery in interproximal intrabony defects with biodegradable barriers. *J Clin Periodontol* 2000; 27: 162–168.
86. Eickholz P, Kim T-S, Steinbrenner H, et al. Guided Tissue Regeneration With Bioabsorbable Barriers: Intrabony Defects and Class II Furcations. *J Periodontol* 2005; 71: 999–1008.
87. Eickholz P, Krigar D-M, Pretzl B, et al. Guided Tissue Regeneration With Bioabsorbable Barriers. II. Long-Term Results in Infrabony Defects. *J Periodontol* 2005; 75: 957–965.
88. Epprecht L, Schlegel C, Holzmann D, et al. Closure of nasal septal perforations with a polydioxanone plate and temporoparietal fascia in a closed approach. *Am J Rhinol Allergy* 2017; 31: 190–195.
89. Gierloff M, Karl Seeck NG, Springer I, et al. Orbital floor reconstruction with resorbable polydioxanone implants. *J Craniofac Surg* 2012; 23: 161–164.
90. Iizuka T, Mikkonen P, Paukku P, et al. Reconstruction of orbital floor with polydioxanone plate. *Int J Oral Maxillofac Surg* 1991; 20: 83–87.
91. Kontio R, Suuronen R, Salonen O, et al. Effectiveness of operative treatment of internal orbital wall fracture with polydioxanone implant. *Int J Oral Maxillofac Surg* 2001; 30: 278–285.
92. Krokidis M, Burke C, Spiliopoulos S, et al. The use of biodegradable stents in malignant oesophageal strictures for the treatment of dysphagia before neoadjuvant treatment or radical radiotherapy: A feasibility study. *Cardiovasc Intervent Radiol* 2013; 36: 1047–1054.
93. Moina DG, Moina G, Rancati A. A Technique to Correct Severe Lateral Crural Concavity: Adjunctive Use of a Polydioxanone Plate During Lateral Crural Reverse Plasty. *Aesthetic Plast Surg* 2014; 38: 1094–1100.
94. Pau M, Reinbacher K, Feichtinger M, et al. Perforating the polydioxanone sheet: Avoiding intraorbital hematoma after open treatment of orbital floor fractures. *J Craniofac Surg* 2012; 23: 1129–1130.
95. Repici A, Pagano N, Rando G, et al. A retrospective analysis of early and late outcome of biodegradable stent placement in the management of refractory anastomotic colorectal strictures. *Surg Endosc* 2013; 27: 2487–2491.

96. Sand JP, Desai SC, Branham GH. Septal Perforation Repair Using Polydioxanone Plates. *Plast Reconstr Surg* 2015; 136: 700–703.
97. Small HN, Braly WG, Tullos HS. Fixation of the Chevron osteotomy utilizing absorbable polydioxanon pins. *Foot Ankle Int* 1995; 16: 346–350.
98. Kalla TP, Janzen DL. Orthosorb: A case of foreign-body reaction. *J Foot Ankle Surg* 1995; 34: 366–370.
99. Chandran P, Kamath RP, Nihal A. Osteochondral fracture of talus treated with bio absorbable pins. *Foot* 2008; 18: 56–58.
100. Prior TD, Grace DL, MacLean JB, et al. Correction of hallux abductus valgus by Mitchell's metatarsal osteotomy: Comparing standard fixation methods with absorbable polydioxanone pins. *Foot* 1997; 7: 121–125.
101. Gill LH, Martin DF, Coumas JM, et al. Fixation with bioabsorbable pins in chevron bunionectomy. *J Bone Jt Surg - Ser A* 1997; 79: 1510–1518.
102. McManners J, Moos KF, El-Attar A. The use of biodegradable fixation in sagittal split and vertical subsigmoid osteotomy of the mandible: A preliminary report. *Br J Oral Maxillofac Surg* 1997; 35: 401–405.
103. Finley DS, Perer E, Eichel L, et al. Ureteral pseudodiverticulum associated with absorbable suture clips after laparoscopic pyeloplasty: case report. *J Endourol* 2005; 19: 726–729.
104. Miller M, Anderson JK, Pearle MS, et al. Resorbable clip migration in the collecting system after laparoscopic partial nephrectomy. *Urology* 2006; 67: 2005–2006.
105. Brusky JP, Tran VQ. Resorbable Clip Migration Resulting in Ureteral Obstruction and Sepsis After Laparoscopic Pyeloplasty. *J Endourol* 2010; 24: 1563–1564.
106. Yasui T, Itoh Y, Maruyama T, et al. The single-knot method with Lapra-Ty clips is useful for training surgeons in vesicourethral anastomosis during laparoscopic radical prostatectomy. *Int Urol Nephrol* 2009; 41: 281–285.
107. Neubauer NL, Schink PJ, Pant A, et al. A comparison of 2 methods of vaginal cuff closure during robotic hysterectomy. *Int J Gynecol Obstet* 2013; 120: 99–101.
108. Kasi Ganesh S, Panneerselvam E, Sharma AK, et al. Knotless Suture for Wound Closure in Intraoral Surgery—A Report of 2 Cases. *J Oral Maxillofac Surg* 2018; 76: 1954.e1-1954.e4.
109. Gupta D, Sharma U, Chauhan S, et al. Improved outcomes of scar revision with the use of polydioxanone suture in comparison to polyglactin 910: A randomized controlled trial. *J Plast Reconstr Aesthetic Surg* 2018; 71: 1159–1163.
110. Gys B, Gys T, Ruyssers M, et al. Laparoscopic Linear Stapled Running Enterotomy Closure in Roux-en-Y Gastric Bypass Using Absorbable Unidirectional Barbed Suture (Stratafix® 2/0). *Obes Surg* 2017; 27: 2740–2741.
111. Obwegeser JA. Osteosynthesis using biodegradable poly-p-dioxanon (PDS II) in Le Fort I-osteotomy without postoperative intermaxillary fixation. *J Cranio-Maxillofacial Surg* 1994; 22: 129–137.
112. Christgau M, Bader N, Felden A, et al. Guided tissue regeneration in intrabony defects using an experimental bioresorbable polydioxanon (PDS) membrane: A 24-month split-mouth study. *J Clin Periodontol* 2002; 29: 710–723.
113. Pretzl B, Kim TS, Steinbrenner H, et al. Guided tissue regeneration with bioabsorbable barriers III 10-year results in infrabony defects. *J Clin*

Periodontol 2009; 36: 349–356.
