# Supplementary material for: Polydioxanone implants: A systematic review on safety and performance in patients
Source: J Biomater Appl. 2019 Nov 26;34(7):902–16. doi: 10.1177/0885328219888841 (PMC7044756; doi:10.1177/0885328219888841)
Supplement: JBA888841 Supplemental Material7 - Supplemental material for Polydioxanone implants: A systematic review on safety and performance in patients [file JBA888841_Supplemental_Material7.pdf]

Supplementary Data 1 - Safety and performance scores for PDO implants found in literature.

Outcomes	Monofilament/Multifilament PDO sutures												
	**Hehl et al.[6866]**	**Murtha et al.[5455]**	**Baracs et al.[3538]**	**Justinger et al.[6934]**	**Diener et al.[3336]**	**Tan-Kim et al.[7067]**	**Brolin[7168]**	**Ulman et al.[7269]**	**Seiler et al.[7370]**	**Bloemen et al.[7471]**	**Albertsmeier et al.[7572]**	**Bassi and Tulandi[7673]**	**Timmermans et al.[7774]**
1. Surgical Site Infection	0.0	3.3	12.5	8.5	15.0	-	0.0	0.0	17.3	7.7	11.3	0.7	13.1
2. Inflammatory reaction	12.0	-	-	-	-	-	-	-	-	-	-	-	-
3. Foreign body reaction	-	19.7	-	-	-	-	-	-	-	-	-	-	-
4. Postoperative fever	-	-	-	-	-	-	-	-	-	-	-	0.0	-
5. Postoperative Pain	-	-	-	-	-	0.0	-	-	-	-	-	-	-
PDO device(s)	PDS™ II	PDS™ II	PDS™ Plus / PDS® II	PDS™ Plus / PDS® II	PDS™ Plus / PDS® II	PDS™ II / Monodek™	PDS™	PDS™	MonoPlus® / PDS™ II	PDS™	MonoPlus® / PDS®	PDS™	MonoPlus®
Number of patients	33	61	485	865	1185	32	120	61	415	233	141	139	107
Unfavourable Outcomes	6.0	11.5	12.5	8.5	15.0	0.0	0.0	0.0	17.3	7.7	11.3	0.4	13.1
**Safety Score**	**94.0**	**88.5**	**87.5**	**91.5**	**85.0**	**100.0**	**100.0**	**100.0**	**82.7**	**92.3**	**88.7**	**99.6**	**86.9**
**Performance Score**	**76.0**	-	-	-	-	**92.6**	-	-	-	-	-	-	**88.8**

Outcomes	Monofilament/Multifilament PDO sutures												
	**Kim-Fuchs et al.[7875]**	**Allahdin, Glazener and Bain[7976]**	**Bayraktar et al.[8077]**	**Breuninger[8178]**	**Cameron et al.[8279]**	**Cassie et al.[8380]**	**Chusak and Dibbell[8481]**	**Constantine et al.[8582]**	**Coras et al.[8683]**	**Deerenberg et al.[8784]**	**Fearon[8885]**	**Gillatt et al.[8986]**	**Varshney, Manek and Johnson[9087]**
1. Surgical Site Infection	0.0	-	19.4	-	8.4	3.6	0.0	-	0.0	22.5	2.8	-	9.4
2. Inflammatory reaction	-	-		8.3	-	-	-	-	-	-	-	-	-
3. Foreign body reaction	-	-		-	-	-	-	-	-	-	-	-	-
4. Postoperative fever	-	-		-	-	-	-	-	-	-	-	-	-
5. Postoperative Pain	16.0	-		-	12.0	-	-	-	-	-	-	-	-
PDO device(s)	PDS™	PDS™	PDS™ II	PDS™	PDS™	PDS™	PDS™	PDS™	PDS™ II and Serasynth®	PDS™ Plus II	PDS™	PDS™	PDS™

Number of patients	133	33	101	665	143	28	52	2	26	545	137	46	100
Unfavourable Outcomes	8.0	-	19.4	8.3	10.2	3.6	0.0	-	0.0	22.5	2.8	-	9.4
**Safety Score**	**92.0**	**-**	**80.6**	**91.7**	**89.8**	**96.4**	**100.0**	**-**	**100.0**	**77.5**	**97.2**	**-**	**90.6**
**Performance Score**	**100.0**	**51.5**	**-**	**93.8**	**99.3**	**-**	**100.0**	**100.0**	**-**	**-**	**97.0**	**82.6**	**94.1**

**Outcomes**	**Monofilament/Multifilament PDO sutures**												
	**Guyuron and Vaughan**[9188]	**Helbling and S**	**Hohenleutner et al.**[9390]	**Hoile**[9491]	**Iwase et al.**[9592]	**Justinger et al.**[9693]	**Justinger et al.**[9794]	**Kasturi et al.**[9895]	**Khan et al.**[9996]	**Kohler et al.**[10097]	**Kreitmann, Riberi and Metras**[10198]	**Leaper et al.**[10299]	**Williams et al.**[103100]

		**chlumpf** 9 2 8 9											
1. Surgical Site Infection	0.0	4.2	0.0	8.5	6.6	10.8	9.2	-	-	26.1	0.0	17.0	12.0
2. Inflammatory reaction	-	-	-	-	-	-	-	-	-	-	0.0	-	-
3. Foreign body reaction	-	-	-	-	-	-	-	-	-	-	-	-	-
4. Postoperative fever	-	-	-	-	-	-	-	-	-	-	-	-	-
5. Postoperative Pain	-	29.2	-	-	-	-	-	-	-	44.1	-	-	-
PDO device(s)	Polydioxanone suture	PDS™	PDS™ II	PDS-C	PDS™ II	PDS™ II	PDS™ II	PDS™	PDS™	PDS™	PDS™	PDS™	PDS™
Number of patients	20	24	126	58	152	1045	409	65	1	81	50	107	100
Unfavourable Outcomes	0.0	16.7	0.0	8.5	6.6	10.8	9.2	-	-	35.1	0.0	17.0	12.0
**Safety**	**100.0**	**8**	**100.0**	**91.5**	**93.4**	**89.2**	**9**	**-**	**-**	**64.9**	**100.0**	**83.0**	**88.0**

**Score**		**3.3**					**0.8**						
**Performance Score**	**91.7**	**95.8**	**92.0**	**-**	**98.7**	**-**	**-**	**83.5**	**100.0**	**81.5**	**100.0**	**98.1**	**-**

**Outcomes**	**Monofilament/Multifilament PDO sutures**													
	**Luciani et al.**[104 ~~101~~]	**Millbourn, Cengiz and Israelsson**[105 ~~102~~]	**Muysoms et al.**[106 ~~103~~]	**Nahas, Augusto and Ghelfond**[107 ~~104~~]	**Nahas et al.**[108 ~~105~~]	**Ohira et al.**[109 ~~106~~]	**Okano et al.**[110 ~~107~~]	**Parara et al.**[111 ~~108~~]	**Ruiz-Tovar et al.**[112 ~~109~~]	**Schenk, Landsiedl and Enenkel**[113 ~~110~~]	**Spencker et al.**[114 ~~111~~]	**Tan, Kannan and Page**[115 ~~112~~]	**Yamaoka et al.**[116 ~~113~~]	**Zhang et al.**[117 ~~114~~]
1. Surgical Site Infection	-	7.8	0.0	-	-	7.4	-	0.0	24.5	-	1.1	-	3.1	0.8
2. Inflammatory reaction	-	-	-	-	-	-	-	-		-	-	-	-	-
3. Foreign body reaction	-	-	-	-	-	-	-	-		-	-	-	-	-

4. Postoperative fever	-	-	-	-	-	-	-	-		-	-	-	-	-
5. Postoperative Pain	-	-	2.0	-	-	-	-	-		-	-	-	-	-
PDO device(s)	PDS™	PDS™ II	PDS™	Polydioxanone Suture	PDS™	PDS™ II	PDS™	PDS™	PDS loop® and PDS Plus loop®	PDS-cord	Polydioxanone Suture	PDS™	PDS™ II	PDS™
Number of patients	181	737	59	10	12	27	11	40	53	13	185	7	323	118
Unfavourable Outcomes	-	7.8	1.0	-	-	7.4	-	0.0	24.5	-	1.1	-	3.1	0.8
**Safety Score**	**-**	**92.2**	**99.0**	**-**	**-**	**92.6**	**-**	**100.0**	**75.5**	**-**	**98.9**	**-**	**96.9**	**99.2**
**Performance Score**	**98.3**	**87.9**	**72.4**	**100.0**	**100.0**	**85.7**	**73.0**	**-**	**86.8**	**94.0**	**100.0**	**-**	**-**	**100.0**

**Outcomes**	**Barbed PDO sutures**											
	**Tan-Kim et al.**[70][67]	**Warner and Gutowski**[118][115]	**Murphey et al.**[119][116]	**Yeo, Lee and Han**[120][117]	**Murtha et al.**[54][55]	**Wright et al.**[63][118]	**Donnellan and Mansuria**[64][119]	**Kelley and Heller**[121][120]	**Liatsikos et al.**[122][121]	**Emanuelsson et al.**[123][122]	**Bogliolo et al.**[124][123]	**Giampa**

												olino et al.[125] ~~124~~
1. Surgical Site Infection	-	0.0	-	-	3.2	33.0	-	-	-	18.0	0.0	4.2
2. Inflammatory reaction	-	-	25.0	0.0	-	-	-	-	-	-	-	-
3. Foreign body reaction	-	-	-	-	10.2	-	-	-	-	-	-	-
4. Postoperative fever	-	-	-	-	-	0.0	0.0	-	-	-	4.2	8.3
5. Postoperative Pain	0.0	-	25.0	-	-	33.0	100.0	-	-	25.0	-	-
PDO device(s)	Quill™ SRS	Quill™	TranQuill	MINT Lift® / Omega 41®	Quill™ bidirectional barbed suture	Quill™ SRS	Quill™	Quil™ SRS	Quill™ SRS	Quill™ SRS	Quill™ SRS	Stratafix®
Number of patients	32	58	20	144	127	3	1	1	6	28	48	17
Unfavourable Outcomes	0.0	0.0	25.0	0.0	6.7	22.0	50.0	-	-	21.5	2.1	6.3
**Safety Score**	**100.0**	**100.0**	**75.0**	**100.0**	**93.3**	**78.0**	**50.0**	**-**	**-**	**78.5**	**97.9**	**93.8**
**Performance Score**	**89.3**	**-**	**45.0**	**97.2**	**-**	**0.0**	**0.0**	**100.0**	**16.7**	**96.4**	**-**	**82.4**

Outcomes	Barbed PDO sutures										
	**Chan et al.**[126 ~~125~~]	**Gililland et al.**[127 ~~126~~]	**Blanc et al.**[128 ~~127~~]	**Gys, Gys and Lafullarde**[129 ~~128~~]	**Kang et al.**[130 ~~129~~]	**Lee, Yoon and Lee**[131 ~~130~~]	**Peleg et al.**[132 ~~131~~]	**Shermak, Mallalieu and Chang**[133 ~~132~~]	**Ting et al.**[134 ~~133~~]	**Yanazume et al.**[135 ~~134~~]	**Zayed et al.**[136 ~~135~~]
1. Surgical Site Infection	0.0	1.0	-	-	-	0.0	-	1.0	0.0	-	-
2. Inflammatory reaction	-	-	-	-	-	-	-	-	-	-	-
3. Foreign body reaction	-	-	-	-	0.0	-	-	-	-	-	-
4. Postoperative fever	-	-	-	-	-	-	-	-	-	-	2.0
5. Postoperative Pain	-	-	-	-	-	-	-	-	-	0.0	-
PDO device(s)	Stratafix®	Quill™ SRS	Stratafix®	Stratafix®	QTL LIFT™	PDO thread	Stratafix®	Quill™ SRS	Quill™	Stratafix®	Stratafix®
Number of patients	55	104	50	100	33	35	51	103	31	20	50
Unfavourable Outcomes	0.0	1.0	-	-	0.0	0.0	-	1.0	0.0	0.0	2.0
**Safety Score**	**100.0**	**99.0**	**-**	**-**	**100.0**	**100.0**	**-**	**99.0**	**100.0**	**100.0**	**98.0**

Performance Score	96.4	-	100.0	60.0	84.8	100.0	68.6	17.5	-	-	96.0

Outcomes	PDO Plates/Meshes										
	Baumann et al.[137136]	Rimmer, Ferguson and Saleh[138137]	Fuller, Levesque and Lindsay[139138]	Petropoulos et al.[5556]	James and Kelly[5758]	Boenisch and Trenité[14059]	Tweedie, Lo and Rowe-Jones[14]	Dayan and Ashourian[141139]	Becker et al.[142140]	Daley et al.[143141]	Dörfer et al.[144142]
1. Surgical Site Infection	-	2.0	2.6	-	1.7	0.0	-	6.7	0.0	-	6.7
2. Inflammatory reaction	3.2	-	-	0.0	-	0.0	-	-	-	-	-
3. Foreign body reaction	-	-	-	0.0	1.7	0.0	2.0	-	-	-	-
4. Postoperative fever	-	-	-	-	-	-	-	-	-	-	-
5. Postoperative Pain	-	-	2.6	-	-	-	-	-	-	-	-

PDO device(s)	PDO Sheets	Perforated PDO plate	Perforated and non perforated PDO plates	PDO foil	PDO foil	PDO plate	Unperforated or perforated PDO plate	PDO plate	PDS foil	Polydioxanone tape	Polydioxanone membrane (Mempol)
Number of patients	31	102	88	12	58	3	50	15	Not clear	45	15
Unfavourable Outcomes	3.2	2.0	2.6	0.0	1.7	0.0	2.0	6.7	0.0	-	6.7
**Safety Score**	**96.8**	**98.0**	**97.4**	**100.0**	**98.3**	**100.0**	**98.0**	**93.3**	**100.0**	**-**	**93.3**
**Performance Score**	**67.0**	**95.1**	**79.5**	**90.9**	**100.0**	**87.5**	**86.0**	**100.0**	**-**	**78.0**	**-**

**Outcomes**	**PDO Plates/Meshes**										
	**Eickholz et al.**[145 ~~143~~]	**Eickholz et al.**[146 ~~144~~]	**Epprecht et al.**[147 ~~145~~]	**Gierlof f et al.**[148 ~~146~~]	**Iizuka et al.**[149 ~~147~~]	**Kontio et al.**[150 ~~148~~]	**Krokidis et al.**[151 ~~149~~]	**Moina, Moina and Racanti**[152 ~~150~~]	**Pau et al.**[153 ~~151~~]	**Repici et al.**[154 ~~152~~]	**Sand, Desai and Branham**[155 ~~153~~]
1. Surgical Site Infection	4.5	3.8	5.0	0.0	0.0	-	-	-	0.0	-	-
2. Inflammatory reaction	-	-	-	-	0.0	6.3	18.2	-	-	-	-

3. Foreign body reaction	-	-	-	-	-	-	-	-	-	-	-
4. Postoperative fever	-	-	-	-	-	-	-	-	-	-	-
5. Postoperative Pain	-	-	5.0	-	-	-	-	-	-	-	-
PDO device(s)	Polydioxanone membrane (Mempol)	Polydioxanone membrane (Mempol)	PDS plate	PDS foil	PDS Plate	PDS plate or sheet	Oesophageal Degradable BD SX-ELLA Stent	PDS plate	PDS sheet	Ella Stent	Polydioxanone plate
Number of patients	21	13	20	194	20	16	11	10	19	11	7
Unfavourable Outcomes	4.5	3.8	5.0	0.0	0.0	6.3	18.2	-	0.0	-	-
**Safety Score**	**95.5**	**96.2**	**95.0**	**100.0**	**100.0**	**93.8**	**81.8**	**-**	**100.0**	**-**	**-**
**Performance Score**	**-**	**-**	**95.0**	**75.0**	**92.6**	**-**	**18.2**	**100.0**	**94.7**	**45.5**	**85.7**

Outcomes	PDO Screws/Pins	PDO Clips/Staples

	Small, Braly and Tullos[156~~154~~]	Kalla and Janzen[23~~25~~]	Chandran, Kamath and Nihal[157~~155~~]	Prior et al. [158~~156~~]	Gill et al. [159~~157~~]	McManners, Moos and El-Attar[160~~158~~]	Finley et al. [43~~46~~]	Miller et al. [161~~159~~]	Brusky and Tran[42~~45~~]	Yasui et al. [62~~64~~]
1. Surgical Site Infection	0.0	0.0	-	0.0	1.7	-	100.0	-	100.0	-
2. Inflammatory reaction	-	50.0	-	-	-	-	-	-	-	-
3. Foreign body reaction	-	50.0	-	0.0	-	-	-	-	-	-
4. Postoperative fever	-	-	-	-	-	-	-	-	100.0	-
5. Postoperative Pain	-	50.0	0.0	0.0	-	-	-	100.0	100.0	-
PDO device(s)	Orthosorb®	Orthosorb®	Orthosorb®	Orthosorb®	Orthosorb®	Orthosorb®	Lapra-Ty® suture clips	Lapra-Ty® suture clips	Lapra-Ty® suture clips	Lapra-Ty® suture clips
Number of patients	71	1	1	39	144	10	1	1	1	30
Unfavourable Outcomes	0.0	37.5	0.0	0.0	1.7	-	100.0	100.0	100.0	-
**Safety Score**	**100.0**	**62.5**	**100.0**	**100.0**	**98.3**	**-**	**0.0**	**0.0**	**0.0**	**-**
**Performance Score**	**84.0**	**50.0**	**100.0**	**95.2**	**-**	**78.0**	**0.0**	**0.0**	**0.0**	**90.0**
