# Supplementary material for: Polydioxanone implants: A systematic review on safety and performance in patients
Source: J Biomater Appl. 2019 Nov 26;34(7):902–16. doi: 10.1177/0885328219888841 (PMC7044756; doi:10.1177/0885328219888841)
Supplement: JBA888841 Supplemental Material8 - Supplemental material for Polydioxanone implants: A systematic review on safety and performance in patients [file JBA888841_Supplemental_Material8.pdf]

Supplementary Data 2 - Safety and performance scores for PDO implants found in literature, when compared to non PDO alternatives.

Outcomes	PDO suture vs non-PDO sutures											
	**Brolin**[7168]	**Ulman et al.**[7269]	**Seiler et al.**[7370]	**Bloemen et al.**[7471]	**Albertsmeier et al.**[7572]	**Gililland et al.**[127126]	**Neubauer et al.**[162160]	**Bassi and Tulandi**[7673]	**Bogliolo et al.**[124123]	**Giampaolino et al.**[125124]	**Chan et al.**[126125]	**Allahdin, Glazener and Bain**[7976]
1. Surgical Site Infection	2	2	0	0	0	0	-	0	-	0	2	-
2. Inflammatory reaction	-	-	-	-	-	-	-	-	-	-	-	-
3. Foreign body reaction	-	-	-	-	-	-	-	-	-	-	-	-
4. Postoperative fever	-	-	-	-	-	-	-	2	0	0	-	-
5. Postoperative Pain	-	-	-	-	-	-	-	-	-	0	-	-
PDO Device(s)	PDS™	PDS™	MonoPlus® / PDS™ II	PDS™	MonoPlus® / PDS®	Quill™ SRS PDO	Monofilament polydioxanone suture	PDS™	Quill™ SRS	Stratafix®	Stratafix®	PDS™
Non-PDO alternative(s)	Ethibond	VICRYL®	VICRYL®	Prolene®	MonoMax®	Ethibond™ / MONOCRYL®	V-Loc 180	V-Loc	VICRYL®	VICRYL®	VICRYL®	VICRYL®
Number of patients in PDO group	120	61	415	233	141	98	58	139	48	17	55	33
**Safety Score**	**2**	**2**	**0**	**0**	**0**	**0**	**-**	**1**	**0**	**0**	**2**	**-**
**Performance Score**	**2**	**2**	**-**	**-**	**-**	**0**	**-**	**-**	**-**	**2**	**2**	**0**

Outcomes	PDO suture vs non-PDO sutures											
	**Breuninger**[8178]	**C a**	**Cassie et al.**[8380]	**Chusak and Dibell**[84 81]	**Ganesh et al.**[163161]	**Gillatt et al.**[8986]	**Gupta et al.**[164162]	**Guyuron and Vaughan**[9188]	**Gys, Gys and Lafullarde**[165163]	**Hohenleutner et al.**[9390]	**Iwase et al.**[9592]	**Justinger et al.**[9693]
1. Surgical Site Infection	-	0	1	2	-	-	-	2	-	2	0	-2
2. Inflammatory reaction	0	-	-	-	-	-	-	-	-	-	-	-
3. Foreign body reaction	-	-	-	-	-	-	-	-	-	-	-	-
4. Postoperative fever	-	-	-	-	-	-	-	-	-	-	-	-
5. Postoperative Pain	-	0	1	-	-	-	-	-	-	-	-	-
PDO Device(s)	PDS™	P D	PDS™	PDS ™	Quill™ PDO	PDS™	PDS®	PDS™	Stratafix®	PDS™ II	PDS™ II	PDS™ II
Non-PDO alternative(s)	Mason 10® and Vicryl and Monocryl®	P r	Nylon	Cat gut suture	VICRYL®	Silk or chromic catgut	VICRYL®	Polyglactin 910 Suture	VICRYL®	VICRYL®	Braided Silk	VICRYL® Plus
Number of patients in PDO group	665	1	28	52	2	46	30	12	100	126	152	1045
**Safety Score**	**0**	**0**	**1**	**2**	**-**	**-**	**-**	**2**	**-**	**2**	**0**	**-2**
**Performance Score**	**0**	**2**	**-**	**2**	**-**	**0**	**2**	**2**	**2**	**2**	**2**	**-**

Outcomes	PDO suture vs non-PDO sutures												
	**Justinger et al.**[97~~94~~]	**Kohler et al.**[100~~97~~]	**Leaper et al.**[102~~99~~]	**Luciani et al.**[104~~101~~]	**Nahas, Augusto and Ghelfond**[107~~104~~]	**Ohira et al.**[109~~106~~]	**Parara et al.** [111~~108~~]	**Peleg et al.**[132~~131~~]	**Ruiz-Tovar et al.**[112~~109~~]	**Zhang et al.**[117~~114~~]	**Ting et al.** [134~~133~~]	**Yanazume et al.**[135~~134~~]	**Zayed et al.**[136~~135~~]
1. Surgical Site Infection	-2	0	0	-	-	0	2	-	-	0	2	-	-
2. Inflammatory reaction	-	-	-	-	-	-	-	-	-	-	-	-	-
3. Foreign body reaction	-	-	-	-	-	-	-	-	-	-	-	-	-
4. Postoperative fever	-	-	-	-	-	-	-	-	-	-	-	-	0
5. Postoperative Pain	-	1	-	-	-	-	-	-	-	-	-	2	-
PDO Device(s)	PDS™ II	PDS™	PDS™	PDS™	Polydioxanone	PDS™ II	PDS II™	Stratafix®	PDS loop® and PDS Plus loop®	PDS™	Quill™	Stratafix®	Stratafix®
Non-PDO alternative(s)	VICRYL® Plus	Dynamesh-IPOM	Nylon	Stainless Steel Wire	Mononylon	Polysorb	Polypropylene and Ethilon and APP	VICRYL® Plus	Vicryl loop® and Vicryl Plus loop®	Silk Thread	VICRYL®	VICRYL®	VICRYL®

							OSE and Vicryl Rapide						
Number of patients in PDO group	409	81	107	181	10	27	40	51	53	118	31	20	50
**Safety Score**	**-2**	**0,5**	**0**	**-**	**-**	**0**	**2**	**-**	**-**	**0**	**2**	**2**	**0**
**Performance Score**	**-**	**-1**	**0**	**2**	**0**	**0**	**2**	**2**	**-1**	**0**	**0**	**-**	**2**

**Outcomes**	**PDO suture vs other non-PDO devices**										
	**Obwegeser**[166164]	**Emanuelsson et al.**[123122]	**Kim-Fuchs et al.**[7875]	**Helbling and Schlumpf**[9289]	**Muysoms et al.**[106103]	**Okano et al.**[110107]	**Schenk, Landsiedl and Enenkel**[113110]	**Spencker et al.**[114111]	**Timmermans et al.**[7774]	**Yamaoka et al.**[116113]	**Shermak, Mallalieu and Chang**[133132]
1. Surgical Site Infection	-	0	2	0	2	-	-	0	0	1	1
2. Inflammatory reaction	-	-	-	-	-	-	-	-	-	-	-
3. Foreign body reaction	-	-	-	-	-	-	-	-	-	-	-
4. Postoperative fever	-	-	-	-	-	-	-	-	-	-	-

5. Postoperative Pain	-	0	0	-	0	-	-	-	-	-	-
PDO Device(s)	PDS™ II	Quill™ SRS	PDS™	PDS™	PDS™	PDS™	PDS-cord	Polydioxanone suture	MonoPlus	PDS™ II	Quill™ SRS
Non-PDO alternative(s)	Titanium miniplates	BARD™ Soft Mesh	Histoacryl	Histoacry l	Polypropylene mesh	Stapling device	Tendon augmentatio n	2-octyl-cyanoacrylate adhesive	Polypropylene mesh	3M™ Skin Stapler	No barbed suture
Number of patients in PDO group	15	28	133	24	59	11	13	185	107	323	103
**Safety Score**	**-**	**0**	**1**	**0**	**1**	**-**	**-**	**0**	**0**	**1**	**1**
**Performance Score**	**0**	**0**	**0**	**0**	**-1**	**-1**	**0**	**2**	**0**	**-**	**0**

**Outcomes**	**PDO Plates/Meshes vs non-PDO devices**							**PDO Screws vs non-PDO devices**		**PDO Clips vs no clips**
	**Dayan and Ashourian**[141~~139~~]	**Becker et al.**[142~~140~~]	**Christgau et al.**[167~~165~~]	**Dörfer et al.**[144 ~~142~~]	**Eickholz et al.**[145~~143~~]	**Eickholz et al.**[146~~144~~]	**Pretzl et al.**[168~~166~~]	**Prior et al.**[158~~156~~]	**Gill et al.**[159~~157~~]	**Yasui**[62~~64~~]
1. Surgical Site Infection	0	2	-	0	-	-	-	2	0	-
2. Inflammatory reaction	-	-	-	-	-	-	-	-	-	-
3. Foreign body reaction	-	-	-	-	-	-	-	-	-	-
4. Postoperative fever	-	-	-	-	-	-	-	-	-	-
5. Postoperative Pain	-	-	-	-	-	-	-	2	-	-

PDO Device(s)	PDS™ plate	PDS foil	PDS membrane (Mempol)	Polydioxanone membrane (Mempol)	Polydioxanone membrane (Mempol)	Polydioxanone membrane (Mempol)	Polydioxanone membrane (Mempol)	Orthosorb®	Orthosorb®	Lapra-Ty® suture clips
Non-PDO alternative(s)	Graft without PDS plate	Collagen membrane	PLA membrane	Polylactide acetyltributyl citrate membrane (Guidor)	Polylactide acetyltributyl citrate membrane (Guidor Matrix Barrier)	Polylactide acetyltributyl citrate membrane (Guidor Matrix Barrier)	Polylactide acetyltributyl citrate membrane (Guidor Matrix Barrier)	K wires / VICRYL®	Kirschner wires	No clips
Number of patients in PDO group	15	Not clear	62	15	21	13	13	39	144	30
**Safety Score**	**0**	**2**	**-**	**0**	**-**	**-**	**-**	**2**	**0**	**-**
**Performance Score**	**0**	**0**	**0**	**0**	**0**	**2**	**0**	**0**	**0**	**2**
